# Optimising in-cell NMR acquisition for nucleic acids

**DOI:** 10.1007/s10858-024-00448-5

**Published:** 2024-08-20

**Authors:** Henry T. P. Annecke, Reiner Eidelpes, Hannes Feyrer, Julian Ilgen, Cenk Onur Gürdap, Rubin Dasgupta, Katja Petzold

**Affiliations:** 1 Department of Medical Biochemistry and Biophysics, Karolinska Institutet, Solnavägen 1, 171 65 Stockholm, Sweden; 2 Department of Medical Biochemistry and Microbiology, Biomedical Center, Uppsala University, Husargatan 3, 752 37 Uppsala, Sweden; 3 Department of Women’s and Children’s Health, Karolinska Institutet, 171 65 Solna, Sweden; 4 Science for Life Laboratory, 171 65 Solna, Sweden; 5 Center of Excellence for the Chemical Mechanisms of Life, Uppsala University, 752 37 Uppsala, Sweden

**Keywords:** In-cell NMR, Longitudinal relaxation rate, Selective excitation, DNA oligonucleotides, Biological replicates

## Abstract

Understanding the structure and function of nucleic acids in their native environment is crucial to structural biology and one focus of in-cell NMR spectroscopy. Many challenges hamper in-cell NMR in human cell lines, e.g. sample decay through cell death and RNA degradation. The resulting low signal intensities and broad line widths limit the use of more complex NMR experiments, reducing the possible structural and dynamic information that can be extracted. Here, we optimize the detection of imino proton signals, indicators of base-pairing and therefore secondary structure, of a double-stranded DNA oligonucleotide in HeLa cells, using selective excitation. We demonstrate the reproducible quantification of in-cell selective longitudinal relaxation times (selT_1_), which are reduced compared to the in vitro environment, as a result of interactions with the complex cellular environment. By measuring the intracellular selT_1,_ we optimize the existing proton pulse sequences, and shorten measurement time whilst enhancing the signal gained per unit of time. This exemplifies an advantage of selective excitation over conventional methods like jump-return water suppression for in-cell NMR. Furthermore, important experimental controls are discussed, including intracellular quantification, supernatant control measurements, as well as the processing of lowly concentrated in-cell NMR samples. We expect that robust and fast in-cell NMR experiments of nucleic acids will facilitate the study of structure and dynamics and reveal their functional correlation.

**Supplementary Information:**

The online version contains supplementary material available at 10.1007/s10858-024-00448-5.

## Introduction

Nucleic Acids (NA) are involved in many cellular processes and have multiple interaction partners (Travers and Muskhelishvili 2015). Most of the current structural biology breakthroughs (Nogales 2015; Carugo and Djinović-Carugo 2023) are performed under in vitro (buffered aqueous solutions or crystals) conditions and not in the native cellular environment. In-cell Nuclear Magnetic Resonance (NMR) has become an important method to investigate protein biophysics and interactions in the cell (Luchinat and Banci 2017, 2023; Yamaoki et al. 2020). Whilst in-cell NMR studies on NA exist, e.g. monitoring structural and stability changes, ligand binding, protein-RNA interaction, and base-pair kinetics inside the cell (Yamaoki et al. 2020, 2018, 2022; Hänsel et al. 2009, 2013; Schlagnitweit et al. 2019; Broft et al. 2021a, 2021b; Dzatko et al. 2020, 2018; Eladl et al. 2023; Viskova et al. 2019; Krafcikova et al. 2019; Cheng et al. 2021; Krafčík et al. 2021), the field can be further developed to enhance the quantitative nature of experiments. Alongside structural understanding of NAs in cells, dynamic behaviour also guides their function and regulation. NMR is uniquely positioned to reveal this structure-dynamics-function relationship (Schlagnitweit et al. 2018; Marušič et al. 2019; Mustoe et al. 2014; Zeng et al. 2014; Alvey et al. 2014; Baronti et al. 2020).

To study dynamic properties of NAs, quantifying the signal intensities is essential and challenging in in-cell NMR, due to the inherent limited spectral quality. Typical spectra display a very low signal-to-noise ratio and have broad line widths, thus in-cell observations are predominantly based on chemical shift information, despite the wide range of NMR parameters and experiments suitable for characterising structure and motion (Broft et al. 2021a). So far, conclusions were mostly drawn by comparing chemical shift data to cell lysate and/or buffered conditions, for example in recent work on structural rearrangements in G-quadruplexes (Bao et al. 2019, 2017; Salgado et al. 2015), DNA ligand binding sites (Krafcikova et al. 2019), ligand binding to RNA aptamers (Broft et al. 2021b) and activity of an antisense oligonucleotide in its native environment (Schlagnitweit et al. 2019).

To tackle the in-cell NMR’s low signal-to-noise ratio, typically one would increase the nucleic acid concentration and/or acquire more data, by increasing the number of scans. However, both strategies are not easily applicable when conducting an in-cell NMR experiment on living human cell lines. Increasing the concentration of the nucleic acid by currently available transfection/translocation methodologies for human cells, e.g. electroporation and streptolysin-O pore formation, is to date limited. Reported concentrations of DNA/RNA, introduced in human cell lines are in the range of 5 to 20 µM (Dzatko et al. 2018) requiring highly sensitive NMR experiments and a large number of scans. The second limitation, the available NMR measurement time, is dependent on both the chemical stability of the target NA and the cell viability. The stability of the target NA can be increased by protection against nucleases, achieved through adding non-native chemical modifications such as phosphorothioate backbones (Hänsel et al. 2009; Yamaoki et al. 2022; Schlagnitweit et al. 2019), and cell viability can be extended via bioreactor setups, recently applied in in-cell NMR of proteins and nucleic acids (Barbieri and Luchinat 2021; Sakamoto et al. 2021). These enhancements are useful, however, it can still be beneficial to keep the in-cell experiment as short and sensitive as possible to observe the nucleic acid before degradation occurs, and to negate the necessity for non-native chemical modifications, to provide an environment with the highest biological relevance. Due to cellular variability, it is also important to consider quantitative replicates in in-cell NMR. Biological replicas are ubiquitous in biological sciences, made necessary by the sensitive nature of experiments, but whilst few studies use them, for example in structure determination or kinetic determination (Gerez et al. 2022; Luchinat et al. 2020), they are not yet pervasive in in-cell NMR.

Development of NMR pulse programs to determine T_1_ is an ongoing process and faster techniques are emerging (Venâncio et al. 2005; Dieringer et al. 2014). There are several approaches to reducing measurement time and/or improving sensitivity, such as the BEST, TROSY or SOFAST schemes (Lescop et al. 2007; Fernández and Wider 2003; Schanda et al. 2005). For each experiment, it is important to set the appropriate parameters to obtain best sensitivity for a given system (Schanda et al. 2005; Diercks et al. 2005). The SOFAST approach combines the benefits of Ernst angel excitation with the enhanced longitudinal recovery following selective excitation. Brutscher and co-workers have shown that there is a dependence of the sensitivity on the recovery time, which is determined by the effective T_1_ of the system (Farjon et al. 2009). The determination of the longitudinal relaxation time T_1_ can be performed with the inversion recovery experiment (180—τ—90—acquisition), in which the signal recovery is recorded as a function of a recovery delay τ (Vold et al. 1968). A selective inversion recovery experiment, which maintains the benefits of enhanced recovery and sensitivity by using a selective pulse, is well suited to in-cell NMR. The selective T_1_ (selT_1_) can be obtained and used to optimise the recovery time, and thus sensitivity, of a SOFAST experiment, alongside any other band-selective NMR pulse sequence. Despite its great advantages, including simplified baseline correction, many nucleic acid in-cell NMR studies on human cells use the per-scan longer acquiring ^1^H Jump-return pulse programs (Krafcikova et al. 2019; Cheng et al. 2021; Dzatko et al. 2018; Hänsel et al. 2013), and the 1D-SOFAST pulse sequence has only recently been used more frequently, although with longer scan times for nucleic acidsfor nucleic acids (Yamaoki et al. 2022; Sakamoto et al. 2021). 2D-SOFAST techniques are common in *xenopus* oocyte studies for nucleic acids and are used for protein in-cell studies (Hänsel et al. 2009; Broft et al. 2021a; Luchinat et al. 2020; Müntener et al. 2016; Barbieri et al. 2016) Both the oocyte and protein in-cell fields generally obtain higher concentration of the biomolecules than nucleic acids in human cell lines (Luchinat and Banci 2017).

Besides optimizing speed and signal intensity, other in-cell NMR controls need to be optimised, e.g. confirmation that the NMR signal originates from inside the cell, and not the extracellular medium. Two main approaches exist, the first in which medium above a cell pellet is used as a proxy for the medium surrounding the cells (Viskova et al. 2019, Sugiki et al. 2020), and the second in which medium surrounding the cells is exchanged through mixing and repelleting (Barbieri et al. 2016; Banci et al. 2013). Although the literature on how to systematically test this control is sparse, spectra of the supernatant control sample are a standard currently in the field and have been shown to be sensitive to the exact experimental procedure (Mu et al. 2017). Identifying the fraction of the unbound species inside the cell in comparison to bound, and thereby undetectable to NMR, species is also important to understand how relevant the observed fraction is.

Here, we establish a pipeline for accurate and reproducible measurements of selective inversion recovery experiments and optimise the delay for the SOFAST experiment on a 12 base pair-long double-stranded DNA (dsA2) (Alvey et al. 2014; Sathyamoorthy et al. 2017), electroporated into Henrietta Lacks (HeLa) cells (CCL-2), and gain a signal enhancement for peaks ranging from 4 to 40% in comparison to jump-return spectra. We implement procedures for important experimental controls, namely the supernatant control to quantify the signal originating from nucleic acids leaked into the interstitial medium, and direct NA quantification, to allow for estimation of nucleic acid transfected into cells. Furthermore, to produce robust and reproducible data, we provide biological triplicates, and explore different processing and analysis approaches. Our supernatant control, microscopy images and flow cytometry data indicate that our DNA sample is approximately equally distributed between the nucleus and cytoplasm in living viable cells, where the recorded selective T_1_ of the imino protons is substantially reduced to 13–30% of the value in the buffered environment.

## Materials and methods

### DNA sample preparation

Single-stranded DNA was purchased from IDT (Sweden) (Strand 1: GCATCGATTGGC, Strand 2: GCCAATCGATGC) (Alvey et al. 2014) and purified by butanol precipitation (Viskova et al. 2019). The dried pellets were either dissolved to 6 mM solution in either: MilliQ water, for in-cell experiments; or 10 mM sodium phosphate buffer (pH 6.5) containing 100 mM NaCl and 0.1 mM EDTA, for in vitro control experiments. Single-stranded DNA was annealed by mixing at a 1:1 stoichiometry and heated to 95 °C using a water bath with a 1 L reservoir, which was then cooled slowly to room temperature. For in-cell experiments the resulting 3 mM double-stranded (ds) DNA solution was mixed with 5’-6-fluorescein (FAM) labelled dsDNA (Integrated DNA Technologies) at 75 µM, and a 1.5 X stock of electroporation buffer (210 mM NaP pH 7.0, 7.5 mM KCl, 15 mM MgCl_2_), and ddH_2_O (Viskova et al. 2019). The final electroporation concentration yielded a 400 µM overall dsDNA concentration of which 2.5% carried a 5’-6-FAM label. In vitro NMR samples of the dsDNA in sodium phosphate buffer were diluted to 1 mM, supplemented with 10% D_2_O.

### Cell preparation and electroporation

We followed a modified protocol based on Viskova et. al. (Viskova et al. 2019) for the preparation of the in-cell sample. HeLa adherent cells (Sigma Aldrich) at passages lower than eight were grown to 90% confluency at 37 °C with 5% CO_2_ in DMEM medium (Sigma Aldrich) supplemented with 10% heat inactivated FBS (Gibco). Cell viability was assessed with Trypan Blue staining and was > 80% preceding experiments (details see Table S1). Approximately 30 confluent 10 cm dishes were harvested by centrifugation (300 xg, 3 min) and washed with 12 mL PBS, from which 100–130 × 10^6^ cells were collected and pelleted. The resulting cell pellet was mixed with 3.2 mL of 400 µM dsDNA in the electroporation buffer, containing 10 µM 5’-FAM labelled DNA. This cell suspension was split into 8–9 Electroporation cuvettes with a 4 mm gap size (Sigma-Aldrich). With 400–500 µL cell suspension per cuvette the samples were chilled for 5 min on ice. Two square wave pulses were applied, first 100 µsec 1000 V high power pulse, interrupted by a 7 s delay followed by a 30 ms pulse with 300 V power using a BTX 830 ECM electroporator (Harvard Bioscience). After incubation at room temperature for 2 min, 500 µl of fresh L15 medium (Sigma) at 37 °C was added and used to wash cells into a larger volume of 42.8 mL. The cells were then washed by centrifugation (300 g, 3 min) and resuspended in 50 mL L15 medium as a final wash. After pelleting again (300 g, 3 min) they were resuspended with 0.6 mL L15 medium with 10% D_2_O. Cells were transferred to a 5 mm Shigemi tube without plunger (BMS-005B, Shigemi Inc., Tokyo, Japan) and a soft ~ 4 cm high cell pellet in the NMR tube was created by gentle hand-crank centrifugation (~ 40 revolutions per minute) (Fig. 6). L15 medium with 10% D_2_O was added to the top of the pellet to 1 cm below the top of the Shigemi tube.

### Flow cytometry (FCM)

500,000 cells were collected on three different time points: (1) before incubation with DNA in the electroporation buffer, (2) after electroporation, but before transfer into the NMR sample tube, and (3) after the first technical replica of NMR measurements. The cells were collected by centrifugation at 300 xg, 3 min, and resuspended in 500 µL PBS. 5 µL of 7-AAD (Tonbo Biosciences) was added and the sample was incubated, sealed from light, for 20 min. The sample was measured with a Beckman Coulter CyAn™ ADP Flow Cytometer and analysed with the Summit Software V4.3. Signal-gates were set to exclude debris and cell clusters.

### Confocal microscopy

The Zeiss LSM 980 confocal laser scanning microscope equipped with a 40 × 1.2 water-immersion objective was used for imaging. The microscope’s objective was additionally provided with a DIC Prism (DIC Prism III PA 63x/1.40, model 426,957, Zeiss, Jena, Germany) for excitation laser depolarization. Hoechst 33,342 (NucBlue Live ReadyProbes Reagent, ThermoFisher) and FAM were excited with 405 and 488 nm lasers, and their emission was detected within the spectral windows of 410–480 and 499–561 nm, respectively. Multitrack mode was used to eliminate the cross-talk between channels. The pinhole parameter was set to 1 Airy unit. The images were taken with four times line averaging.

### NMR measurements

All NMR experiments were performed using a Bruker Avance III HD spectrometer operating at 600.16 MHz ^1^H base frequency equipped with a 5 mm QCI (^1^H/^13^C/^15^N/^31^P) cryoprobe with z-gradient (50 Gs/cm maximum gradient strength). NMR spectra were acquired and processed using Bruker Topspin 3.6 software. All experiments were carried out at 298 K. Tuning, matching and shimming was done manually. All gradients had a sine-bell shape (SINE.100) and were digitized using 100 points. The gradient strengths are given as fractions of the maximum amplitude and are followed by a recovery delay (D16) of 200 μs. For all samples under investigation the ^1^H 90° pulse was manually calibrated before running further experiments.

### 1D ^1^H-SOFAST experiments

All 1D SOFAST spectra, to test the quality of each freshly prepared in-cell sample, were acquired using a modified SOFAST-HMQC sequence (Schanda et al. 2005). Briefly, the pulse sequence contains a selective excitation pulse followed by a gradient-selected selective spin-echo with a selective refocusing pulse and signal detection. Selective excitation of the imino protons was performed using a PC9 (*Pc9_4_120.1000*) (Kupše and Freeman 1994) with a length of 3.88 ms (3 ppm bandwidth) and 120 excitation angle. The selective refocusing was done with a 3.23 ms long Reburp pulse (*Reburp.1000*, 3 ppm bandwidth)(Geen and Freeman 1969). Power calculation of the selective pulses is hard-coded in the pulse sequence based on the duration and power level of the hard ^1^H 90° pulse (8.6 W). The carrier frequency was set for both selective pulses to the center of the imino region at 13.5 ppm. Gradients for coherence selection in the selective echo as well as a purging gradient before excitation had durations of 1 ms and strengths of 11% and 7%, respectively.

The spectra to test the quality of each freshly prepared in-cell sample were acquired with 2048 scans using a recovery delay D1 = 30 ms and an acquisition time of 300 ms giving a total experiment time of 13 min. The SOFAST spectra to show the signal-to-noise in comparison to the jump-return experiment of same total experimental time were acquired with 9216 scans using a recovery delay D1 = 30 ms and an acquisition time of 77 ms giving a total time of 23 min and 37 s.

Benchmark test for in vitro experimental evaluation of optimal recovery time performed as above with acquisition time of 77 ms and 1024 scans, on a sample of 150 µM dsDNA. Recovery delay times (D1) 0.0305, 0.06, 0.12, 0.15, 0.18, 0.2, 0.24, 0.27, 0.3, 0.33, 0.36, 0.39, 0.42, 0.45, 0.48, 0.51, 0.54, 0.57, 0.6, 0.63, 0.66, 0.69, 0.72, 0.75, 0.422, 0.43, 0.44, 0.45, 0.46, 0.47. Fits and plots were performed using Eq. (1) (Schanda et al. 2005):

1 $$\frac{\text{sin}\left(\frac{\pi }{3}\right){*(1-\text{exp}(-T}_{rec}/{selT}_{1})}{(1-\text{exp}\left({-T}_{\mathit{rec}}/{\mathit{selT}}_{1}\right)\text{*cos}\left(\frac{\pi }{3}\right))*\sqrt{T_{scan}}}$$

With T_rec_ = recovery time (recovery delay + acquisition time), selT_1_ = selective T_1_, T_scan_ = time for one scan (recovery time + pulse lengths).

### 1D ^1^H jump-return experiments

The 1–1 jump and return experiment (Guéron et al. 1969) was performed with an acquisition time of 116 ms and a recycle delay of 1.2 s. This ranges from 1.3x (T9) to 6x (G11)*nonselT1. The offset for the delay was placed at 13 ppm while the carrier frequency was at water resonance (4.7 ppm). The signal was averaged over 1024 scans with a spectral window of 22.04 ppm giving a total experimental time of 23 min and 22 s.

### In-cell selective inversion recovery experiment

Our experiments are divided into biological replicates, which are individual in-cell experiments performed at different days, and technical replicates, which are repeated NMR measurements on the same sample. The pulse sequence to determine the selective longitudinal relaxation time (selT_1_) contains a selective inversion pulse, followed by a selective excitation pulse with a short filter gradient in between. For better water suppression a gradient-selected selective spin-echo was applied between selective excitation and signal detection. For selective inversion and excitation a 1.3 ms long Iburp pulse (*Iburp2.1000*, 5.8 ppm bandwidth) (Geen and Freeman 1969) and a 1.4 ms long Eburp pulse (*Eburp2.1000*, 5.8 ppm bandwidth) (Geen and Freeman 1969) were used, respectively, whose carrier frequency were set to 15 ppm. The selective refocusing was done with a 4 ms long Reburp pulse (*Reburp.1000*, 2.4 ppm bandwidth) (Geen and Freeman 1969), whose carrier frequency was shifted by −2 ppm to the center of the imino region (13 ppm). This selective pulse setting was chosen for reduced baseline distortions in the proton region of interest. Gradients for coherence selection in the selective echo as well as a purging gradient before the selective inversion pulse excitation had durations of 1 ms and 1.5 ms and strengths of 40% and 17% respectively. The short purging gradient in between the selective inversion and excitation had a duration of 100 μs and a strength of 50%.

The acquisition time was 310 ms with a D1 of 658.5 ms, giving a total recovery time of 968.5 ms, which is expected to match ~ 3*(non-selective)T_1_ which corresponds to 95% magnetisation recovery estimated from an in vitro sample. We recorded 1024 scans for each delay τ, giving total experimental times per selective inversion recovery time point of 17 min and 34 min for the shortest and longest delay, respectively. Nine delay times were measured for one recovery curve: 0.320, 150, 300, 600, 1200, 0.320, 225, 450 and 900 ms. This series represents one technical replicate, of which we recorded three technical replicates on one biological replicate. 0.320 ms was repeated and used to identify sample deterioration through a linear fit of the signal decay. Additionally, we recorded a biological replicate with shorter delays to better characterise the first points in the recovery curve, as the initial biological replicates showed that the selT_1_ was shorter than anticipated. At 0.320, 15, 50, 250, 900, 0.320, 7.5, 75, 4 ms. The first set of τ was recorded in 2 h and 2 min, while the second set was recorded in 1 h and 39 min. After the first technical replicate, we prepared a supernatant control (see next section and Fig. S1). For each in-cell sample, we recorded three technical replicates of the inversion recovery experiment over the course of 12 h.

### Supernatant sample preparations

Two approaches for supernatant preparation were used. For biological replicate 2 and 3 we removed all media from the cells and then the cells from the Shigemi tube. Their volume was measured and then they were pelleted at 300 xg for 3 min in a 1.5 mL Eppendorf tube. The supernatant was collected. The cells were then resuspended in a volume of medium corresponding to: volume of cells minus the volume of first supernatant. They were then pelleted again at 300 xg and the two supernatants combined. For biological replicate 1 and the final jump-return and SOFAST comparison experiment we used the following approach. The supernatant NMR sample is created by the following steps (Fig. 6): (1) measure the height of the active volume of the pellet V_P_ on the Shigemi wall, (2) The medium above the cell pellet is removed in two steps, (a) V_Excess_, which is furthest away from the pellet, and (b) V_1_, which is the volume right above the pellet and equal in volume to the cell pellet volume itself. V_1_ is removed using a Pasteur pipette (3). V_1_ is added back to the pellet and the cap is placed on the Shigemi tube. The Shigemi tube is then gently inverted back and forth until the pellet has been resuspended (4) the cells are pelleted again by hand centrifuge and (5) the remaining medium V_1_ is aspirated carefully to not shear or aspirate cells. (6) The supernatant V_1_ is transferred into a new Shigemi tube and produces the supernatant control. (7) Fresh L15 medium V_New_ supplemented with 10% D_2_O is placed in the Shigemi tube above the cell pellet.

### Estimating volume of medium surrounding pelleted cells

Cell volume was calculated by estimating the average diameter of cells from microscopy, assuming a spherical shape. 100 million cells (in DPBS) were centrifuged at 300 xg for 3 min., and the medium was then carefully aspirated. Cell pellet was then resuspended in 600 µL and the final suspension volume was measured. The volume of medium surrounding the cells could then be estimated by subtracting a total cell volume of 2.5 pL per cell, calculated as above.

### Data analysis

The series of spectra from the inversion recovery experiment was processed using TopSpin 3.6 (Bruker, USA) self-written scripts (available on github.com/PetzoldLab/T1-in-cell). The chemical shift of the peak maxima was fixed to the value as observed in the first delay time, and was used to identify the peak intensities for each spectrum. In vitro data of the selective inversion recovery experiments were recorded to quantify the selT_1_ in an aqueous NMR buffer on a 1 mM sample. In-cell NMR spectra were processed in three alternative ways. The first with an exponential window function EM with applied line broadening of 1 Hz, the free induction decay (FIDs) were additionally truncated from 2048 to 512 (TD_eff_ = 512). In the second approach, we applied the same processing, with an additional linear prediction with 16 coefficients and 8192 output points (Dickson et al. 2022). In the third approach, no truncation was applied, and a line broadening of 15 Hz was used. We calculated the standard error for the peak intensities by I / (SNR* 2), which extracts the RMS (root-mean-squared) noise (SNR = signal-to-noise). The signal-to-noise ratio value for each spectrum was calculated with the SINO command in Topspin, setting the noise area from 15 to 16 ppm in the ^1^H spectrum. Care must be taken to make sure that there is no systematic raised baseline, which will falsify the SNR. Data fitting was performed with Origin (Origin Lab, MA, USA). The absolute intensities at delay τ were fitted globally to the mono-exponential recovery equation $I=D{e}^{-\tau /sel{\rm T}1}+A$ sharing parameter T_1_ (with D = Coefficient for correction of imperfect inversion, A = equilibrium intensity, τ = variable recovery delay, selT_1_ = relaxation time). We plotted the normalized peak intensities by dividing the absolute intensities by the fitted parameter A, which represents the maximum peak intensity at infinite delay time τ.

### Subcellular quantification

Cell and nuclear diameters (n = 10) were taken from confocal microscopy image (Fig. S2, middle panel), from which volumes were calculated assuming a sphere to be representative. The fluorescence intensity inside the nucleus and cytoplasm was measured and approximated as a fluorescence density in order to obtain the total fluorescence of a cell and nucleus of a certain volume. All image analysis performed in Fiji.

### Quantification of transfected in-cell DNA concentration by denaturing PAGE

Cells were taken either before electroporation, whilst in PBS, or after electroporation once in the final L15 10% D_2_O medium and frozen on dry ice. The cells were lysed by addition of PAGE gel loading dye containing 64 mM EDTA, 388 µM Bromophenol Blue dissolved in Formamide (Karlsson et al. 2020), and subjected to three freeze–thaw cycles. The samples were loaded onto a 20% denaturing PAGE (d-PAGE), containing 8 M Urea (Sigma-Aldrich) and TBE buffer (Thermofisher Scientific) and 20% acrylamide (Bio-Rad), preheated for 15 min and then run at 350 V for 1 h alongside standard curves, prepared from 5′ 6-FAM 12mer dsA2 (IDT) dilutions. The gel was imaged (Fig. 2, original gel attached) using ImageQuantTM LAS 4000 Cy2 filter (460 nm blue light and Y515 Di filter) with 30 s exposure. For quantification, ImageLab (Bio-Rad) was used and the background autofluorescence from untransfected HeLa cells was subtracted. The standard curve was plotted and fitted using Prism (GraphPad). Once a quantity of fluorescent dsDNA was found for a given number of cells, the quantity is multiplied by the ratio of fluorescent and non-fluorescent dsA2 (1:40), then divided by the total number of cells, with background subtracted. HeLa cell volume was assumed as previously calculated (2.5 pL) (see Materials and Methods section Confocal Microscopy).

## Results

### Fold preserved in the cellular environment

**Fig. 1:**
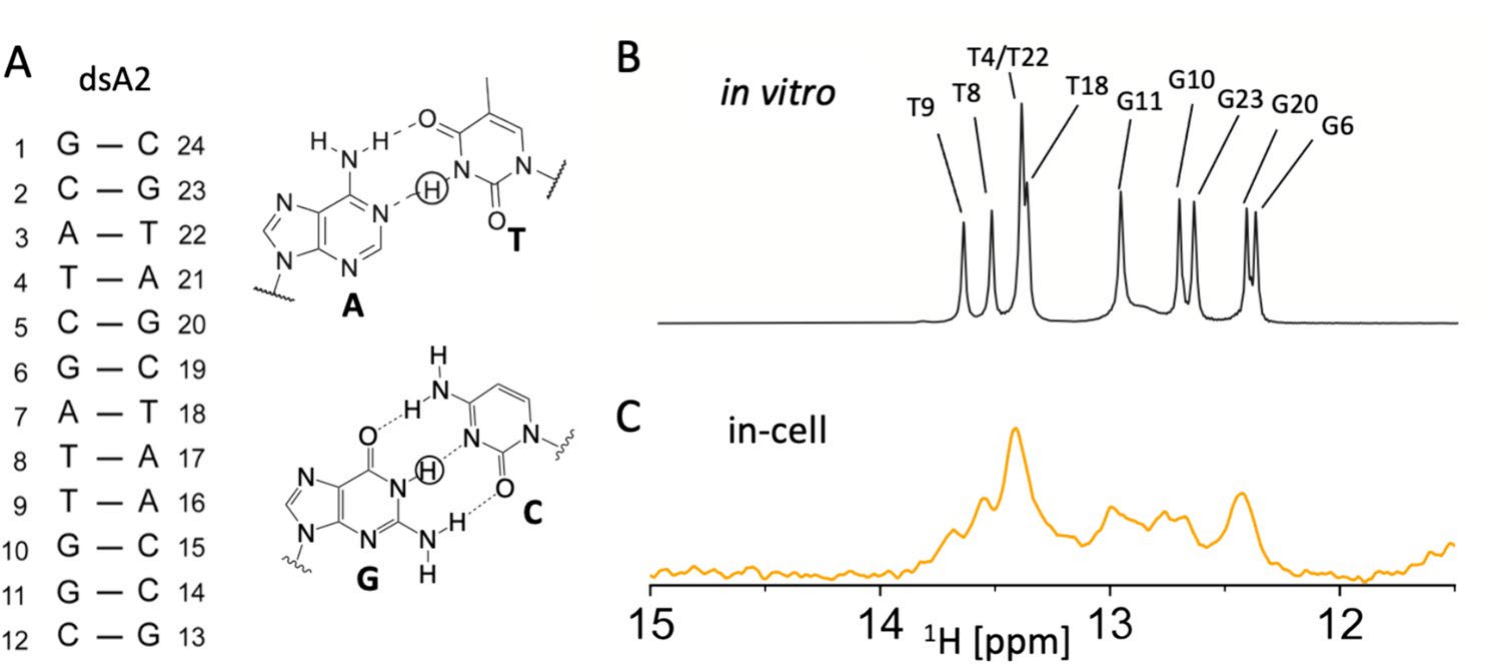
12mer dsA2 profile **A**: secondary structure of the dsA2 alongside relevant base pairings visible in the imino region (~ 10–15 ppm) with the imino protons detected indicated by circles. **B**: in vitro jump-return spectrum of the 1 mM dsA2 in NMR buffer. **C**: In-cell Jump-return experiment acquired with 1024 scans (23 min 29 s) of electroporated HeLa cells containing dsDNA

In-cell NMR measurements of a short dsDNA duplex (dsA2) in HeLa cells showed that all base pairs were maintained in the cell, making it a suitable candidate for selT_1_ studies comparing in-cell to in vitro. The dsA2 (Fig. 1A) (Alvey et al. 2014), was selected for the in-cell NMR studies because of its size (12 mer), dispersed NMR signals in the imino region and well characterized structure and dynamics (assignment (Sathyamoorthy et al. 2017), Fig. 1B). It consists of a smaller two base pair long AT stretch (A2), as longer AT- regions could potentially interact with cellular milieu (Haran and Mohanty 2009; Patsialou et al. 2005). Figure 1C shows that the imino signals from the dsA2 could be detected using a conventional jump-return NMR pulse scheme (Guéron et al. 1969). Although the jump-return spectrum from in-cell spectra matches the profile from in vitro spectra (Fig. 1B) resonances from iminos are broadened, preventing resolution between T4, T22 and T18, and G20 and G6. This indicates, however, that the basic structure in cells is the same as detected in vitro.

### Cellular quantification

**Fig. 2:**
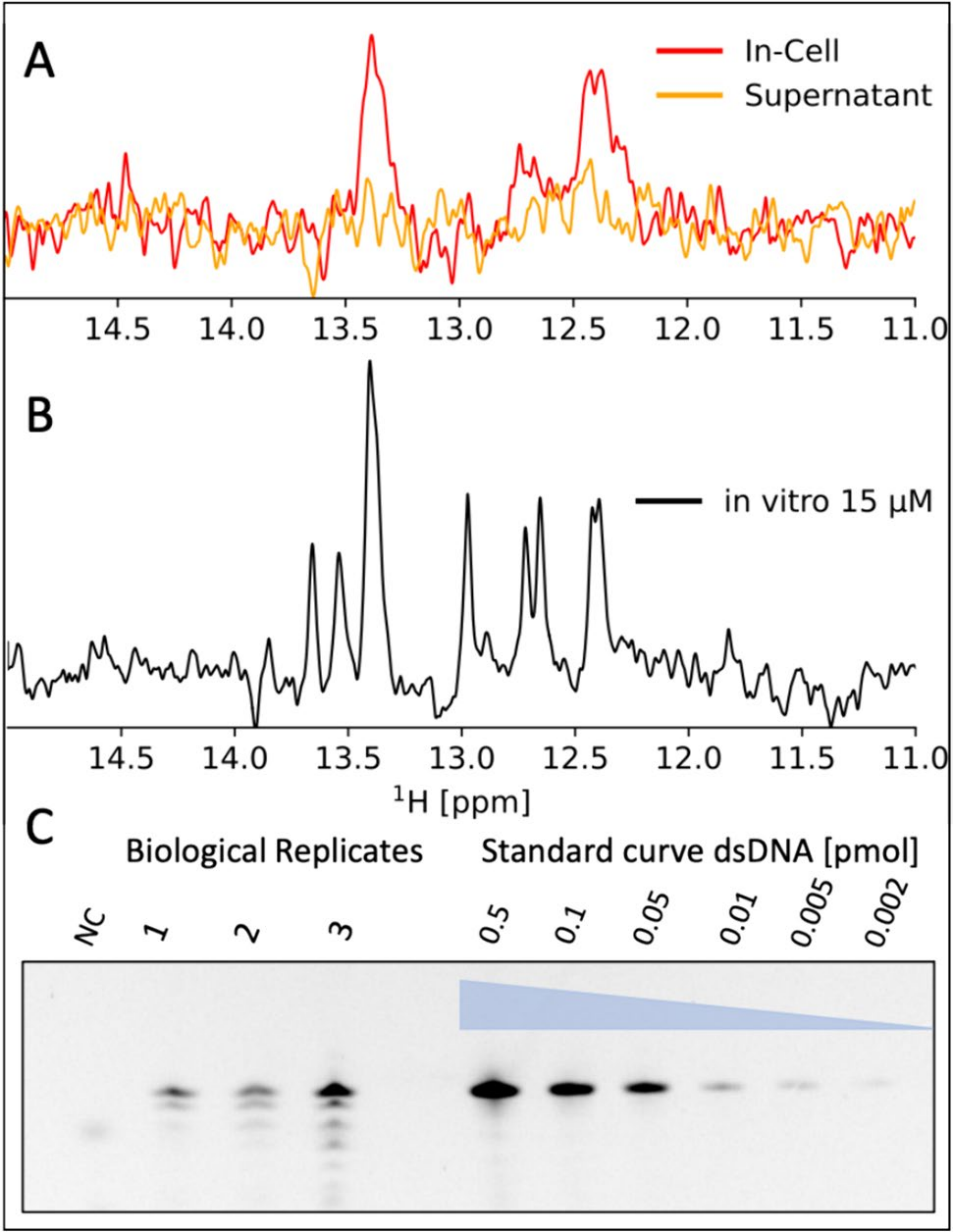
Signals observed arise from the free dsDNA species present inside the cell. **A**: SOFAST-1D spectra acquired with 2048 scans (13 min) in-cell (red) overlayed on the supernatant control (orange), indicating the signal originates from inside the cell. **B**: For comparison the 15 µM dsA2 recorded with the same parameters in vitro*.* **C**: 20% d-PAGE of cell lysates alongside standard curve imaged with FAM fluorophore labelled dsDNA, in which 2.5% DNA was labelled with FAM, to estimate total cellular concentration of dsDNA

Quantification of the intracellular concentration of the introduced nucleic acids reveals that the majority of dsA2 inside the cell is not bound to larger cellular complexes, as the presence of NMR signal is dependent on the free mobility of molecules. Using the FAM-labelled dsA2 to quantify the total amount in the cell lysate on a d-PAGE, we can show that the NMR-detectable fraction is comparable to the total amount of introduced dsA2 in the cell through electroporation (Fig. 2). The concentration of dsA2 inside the cell can be estimated by including standard curves of FAM-labelled dsA2 (linear correlation fitted for the standard curve Fig. S3, R^2^ = 0.99). The highest concentration of 0.5 pmol was omitted as it lies outside the linear range of the detector, likely saturating the detector, while all the in-cell data points lie within. An estimation of the cellular volume is needed, and therefore the diameter of n = 10 cells from Fig. S2 were measured, and the cell volumes were approximated as spherical. This resulted in 17.0 ± 7.2 µm with a volume of 2.5 ± 0.5 pL. With minimal purification steps, to reduce sample loss, we quantified the cellular concentration to be 13.6 µM ± 4.0 µM, as an average over three biological replicates (Fig. 2, Fig. S3). Our in-cell spectra are similar to those of an in vitro sample of 15 µM, and integrating the imino peaks reveals only 5% larger integrated intensity in-cell. It is important to note that integrating between sample compositions with different shimming and magnetic susceptibility means that this serves only as an approximation. This would, however, indicate that most of the introduced dsA2 remains free from larger complexes, as it is visible – indicative of free tumbling (Fig. 2), albeit with reduced resolution, which can be a result of transient interactions with larger molecules, interactions with smaller molecules, structural or sample inhomogeneity and/or different shimming properties.

### Cellular viability

High viability and transfection efficiency could be determined by two complementary cell viability assays and fluorescent labelling of dsA2. Flow cytometry data indicates that the dsDNA is efficiently transfected by electroporation (Fig. S4) as 83 ± 11% of all cells carry FAM-labelled dsA2 and are viable before the NMR measurements starts. Interestingly, a discrepancy is noticeable between the viability assays, Trypan Blue microscopy viability and flow cytometry, which could arise from the settings of the signal gates, or different properties of the staining dye (Table S3). Trypan Blue staining has been shown to have different biases compared to other viability stains (Mascotti et al. 2000). This is an indication of the importance of orthogonal methods for controls, especially for fields where methods are being newly established like in-cell NMR of nucleic acids.

### Subcellular localisation

Although there is a visibly higher concentration of the labelled dsA2 inside the nucleus, calculations reveal that the two molecular counts are closer to equal, due to the cytosolic volume being larger. We performed confocal microscopy on our electroporated cells and could detect that the dsA2 is distributed in both cytosol and nucleus, with visibly higher concentrations in the nucleus (Fig. S2). Upon quantification, using a nuclear diameter average of 9.6 ± 2 µm, it was revealed that 18.0 ± 25.0% of the cellular volume is nuclear, in line with other findings ranging from 1–20% (Wu et al. 2022; James and Giorgio 2000; Cadart et al. 2018), alongside observations that this value can vary within populations due to cell cycle stages or environmental effects (Moore et al. 2019; Franklin et al. 2020; Kemper et al. 2007). With a concentration ratio of 65:35 (± 7%) between nucleus and cytoplasm, this reveals that the fluorescence intensity arising from the nucleus contributes 30 ± 29% of the total signal.

### dsA2 outside of cells during sample preparation is only removed upon washing

By measuring the RNA concentration in the volume of interstitial fluid in pelleted cells, it is revealed that a singular washing step leads to NMR-detectable quantities of dsA2 in the surrounding medium. Washing of the cells after electroporation needs to be sufficient to remove excess dsA2 and prevent unwanted signals before the NMR sample is prepared. In order to determine this, we estimated the medium volume surrounding cells pelleted at 300 g for 3 min by resuspending 100 million cells in in 0.6 mL of L15. The resulting volume (1.07 mL) is the sum of cell volume and interstitial medium. We estimated the HeLa cell volume to be 2.5 ± 0.5 pL, by using the diameter average from microscopy images (n = 10), (Fig. S2B). From the estimated cell volume of 100 million cells (250 µL) and the precise measurement of the total volume of a cell pellet through measurement of the volume change (0.47 mL), it was concluded that ~ 46% of the cell pellet contains medium (n = 1). This means that after washing only once in 15 mL after electroporation of 100 million cells in 400 µM dsA2, followed by resuspension in 0.6 mL medium, one would expect that ~ 1.9 µM dsA2 remains in the supernatant, just by the dilution effect. To avoid this source of error we include two washing steps with 50 mL of L15 medium following electroporation, leading to a theoretical remaining concentration of 0.002 µM dsA2.

### Measurement of selective T_1_ using selective inversion recovery

**Fig. 3:**
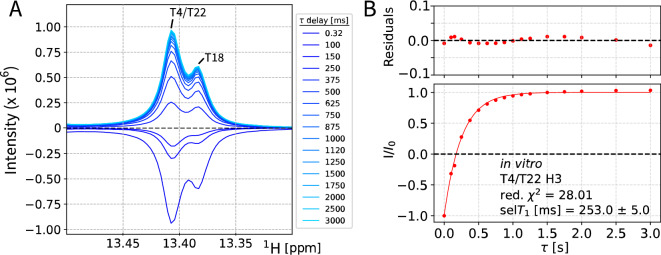
In vitro estimation of selT_1_ by selective inversion recovery. **A**: recorded with the selective inversion recovery pulse sequence using an in vitro 1 mM dsA2 sample in NMR buffer. Each point represents a selective inversion recovery experiment using recovery delay τ and 32 scans, resulting in experiments ranging from 6 min 3 s to 8 min 3 s. The remaining signal is T18 and the fit is shown in Fig. S5. **B**: Exponential fit with I = A + D*exp(-τ/selT_1_) of T4/22 intensities. Values normalized to the estimated equilibrium intensity. The fitted selT_1_ (T4/T22/T18; in vitro) is 253 ± 5 ms

To quantify the selT_1_ of T4/22 and T18 in vitro, alongside all other visible peaks, we recorded an inversion recovery experiment with selective excitation pulses sampled from 0.32 to 3000 ms (Materials and Methods) (Fig. 3). Data fitting was performed using an approximation of a mono-exponential recovery fit (Farjon et al. 2009; Szulik et al. 2014; Guéron and Leroy 1995).

T18 and T4/T22 share values in the same range 224 ± 4 ms and 253 ± 5 ms. The in vitro recorded selT_1_ are in the range of 107 – 330 ms (Table S1), which agrees with the range of reported values (at lower temperatures for Kearns et. al.) (Farjon et al. 2009; Behling et al. 1984).

### selT_1_ reduced in the cellular environment

**Fig. 4:**
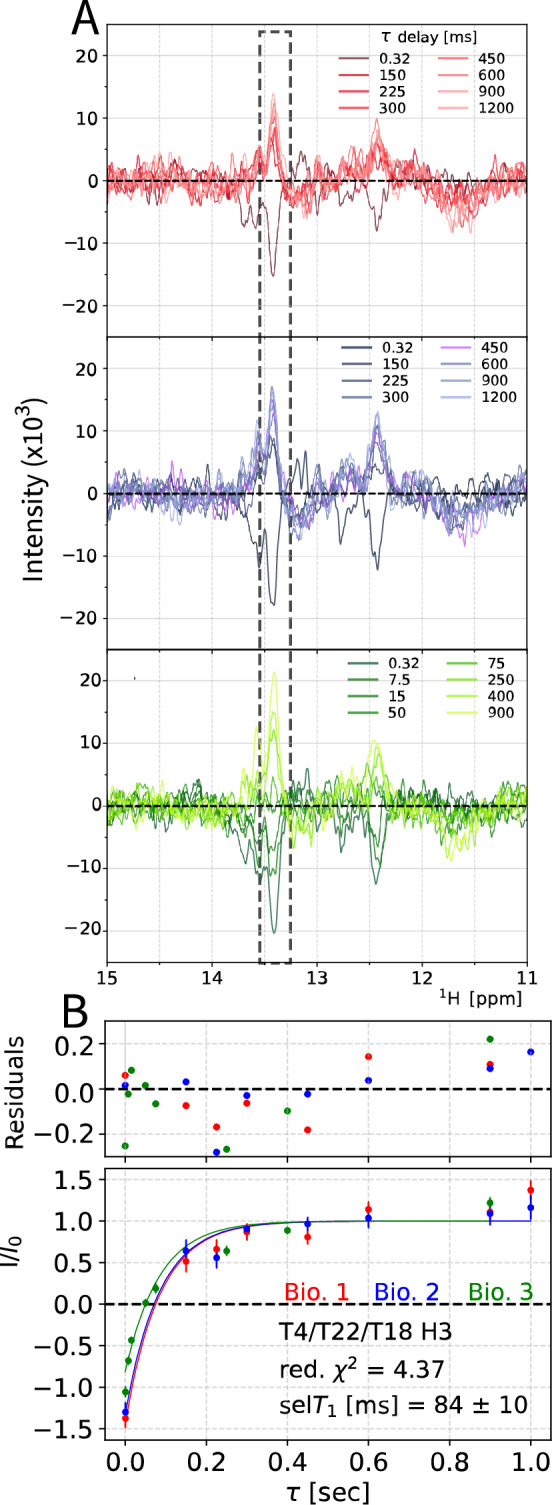
Determination of selT_1_ in HeLa cells. **A**: Three biological replicates of the selective inversion recovery experiment of dsA2 in HeLa cells, recorded at 298 K. Top to bottom is first to third replica, respectively. **B**: Global exponential fit with shared parameter of selT_1_ from the three biological replicates within the first two hours of measurement, the first technical replicate (see Fig. S6 for all spectra and Fig. S7, S8 for all fits). Exponential fit with I = A + D*exp(−τ/selT_1_). Values normalized to the signal at equilibrium

**Fig. 5:**
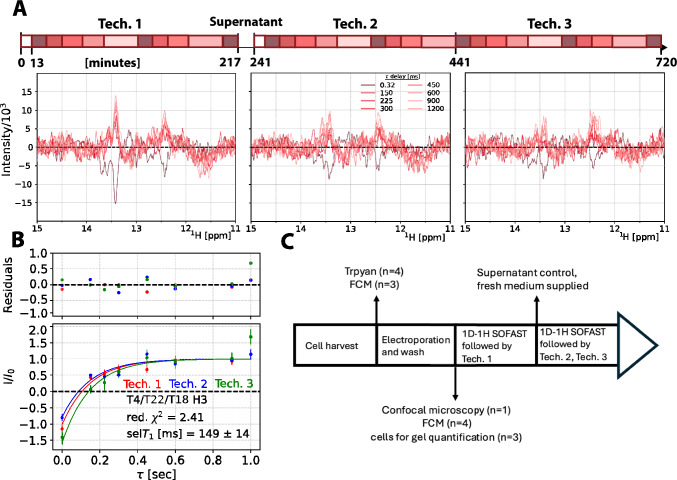
Global analysis of selT_1_ of first technical replicates over three biological replicates (**A**). Three technical replicate spectra of biological replicate 1 (other replicates in Fig. S8), individual spectra coloured in a gradient based on delay τ. The schematic time arrow above represents the start time of a spectrum over the course of the measurement. White boxes represent SOFAST-1D spectra acquired with 2048 scans (13 min) (**B**). Global fits of exponential recovery for selT_1_ (Eq. 1) of the three technical replicates. **C** General workflow for the selective inversion recovery experiment from sample preparation to measurement, including experimental controls

By measuring selective inversion recovery measurements on dsA2 in-cell it was observed that the selT_1_ is reduced in the cellular environment. To measure our selective in-cell selT_1_, we recorded the experiment on three biological replicates (Fig. 4A), each consisting of three technical replicates, Fig. 5, corrected for sample deterioration. Human cells can exhibit markedly distinct behaviours under only slight variations in conditions, such as changes in the cellular growth medium, hence the requirement for biological triplicates arises (Moore et al. 2019; Franklin et al. 2020).

Recovery curves were fit to all imino peaks, excluding G23/G6 (12.6 ppm), for which the peak maxima observed shifted significantly between experiments, resulting in failed fits for all experiments. This was likely due to the peaks being more separated than others, but not resolved enough to identify individual peaks. The most intense imino proton signal in our in-cell spectrum of dsA2 arises from the triple T4/22/18 peak at 13.4 ppm. Our measurements represent an average of the three.

At the low signal-to-noise ratios observed in-cell, we controlled for potential biases in each step of analysis: processing, extraction, and fitting (Kemper et al. 2007). We processed the data in three different ways: simple line broadening, FID truncation, and FID truncation with linear prediction (Fig. S9). It was observed that truncating the FID maintained sufficient resolution and led to more consistent peak maxima in chemical shifts by reducing noise, which indicates that the acquisition time was longer than necessary (Fig. S9), however, sufficient acquisition time is required for quantitative assessment of selT1, to allow for signal recovery between scans. By comparing three different processing methods and arriving at the same selT_1_ for the lowest intensity peak T9, we gained further confidence in the data processing methods (Fig. S9). Furthermore, we chose to fix the peak maxima at a given chemical shift, as indicated by Viles et al. (Viles et al. 2001).

**Fig. 6:**
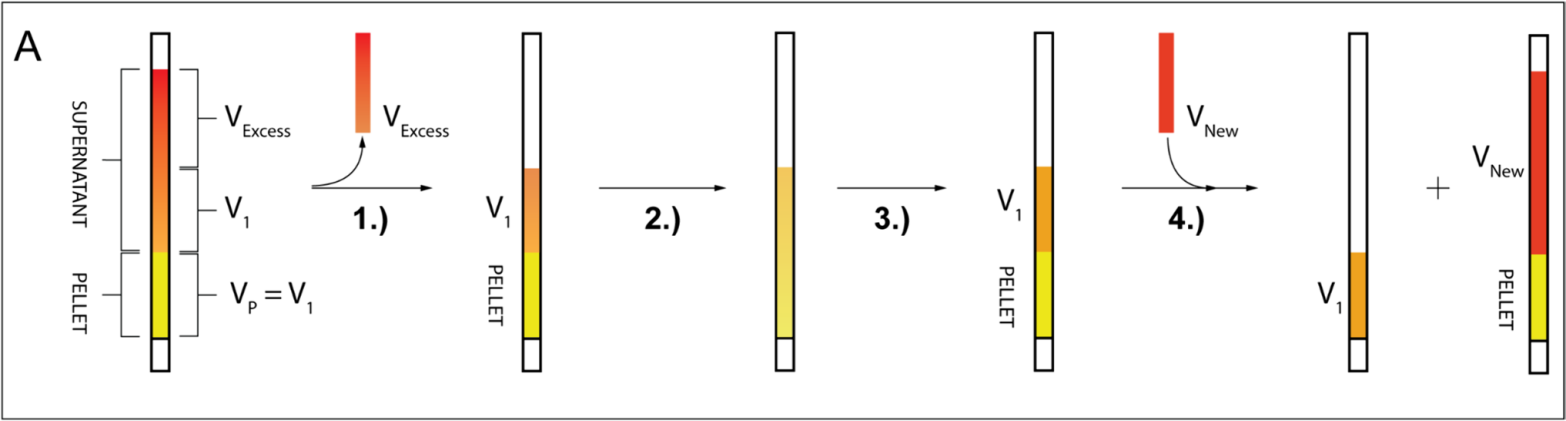
Workflow of the gentle supernatant sample procedure: The in-cell sample (PELLET) was roughly the size of the NMR-probe coil (V_P_), called “active volume”. (1) Excess medium V_Excess_ was discarded. (2) V_1_ was aspirated with a Pasteur pipette and the cell pellet is resuspended by flushing V_1_ one time on the pellet and then repeatedly inverting the sample tube until the sample is homogeneous (3.) The cell pellet was formed by hand crank centrifugation. (4.) The supernatant V_1_ forms the supernatant NMR sample which contains only leaked DNA from the interstitial medium and was placed in a new sample tube. The washed cells were layered with a V_Excess_ equivalent volume of fresh L15 medium

Three series of recovery curves (technical replicates) within each biological replica were recorded (Fig. 5). This allowed for three different fit analyses. Individual technical replicate fitting, global fitting over all technical replicates of one biological replicate, and global fitting over all biological replicates. First, each technical replicate was fit individually (Fig. S8, Table S2), and renormalised by remeasuring the 0.32 ms timepoint. Technical replicates allowed for the observation of sample change over the course of a measurement. Due to increasing fit uncertainty over the course of the measurement, there is no consistent change in the selT_1_ observed over the total 12 h. Second, it was possible to globally fit all technical replicates from one biological replicate (Fig. 4). The selT_1_ value of each replicate is compensated for sample deterioration following sample degradation and cell death (from 84% viability to 69% after 4 h), by fitting linear decay values to interleaved 0.32 ms experiments. This tracked sample decay and allowed for its correction, Fig. S10. Cell death starts after electroporation as cell viability drops from 97 ± 4% to 89 ± 9% and can be expected to further decrease during the inversion recovery NMR measurement. After 4 h (one technical replicate) of measurement the viability reduces by another ~ 4%. The global fitting is superior to individual fits, confirming common physical parameters observed in each experiment, however, the inclusion of data acquired long after sample preparation, for example technical replicate 3, is suboptimal due to the decrease in viability (Table S2). The final chosen approach involves globally fitting the first technical replicates between the three biologically replicates. This has the advantage of being acquired when the cells are closest to their physiological state, increasing the data points in this time window, alongside allowing for different delay times to be used to fully characterise the recovery.

By globally fitting the first technical replicate (spectra acquired within the first four hours of measurement) of the three biological replicates, a selective T_1_ (T4/T22/T18; in-cell, Tech. rep. 1, global) = 84 ± 10 ms is fitted corresponding to 1/3 of its in vitro value of 253 ± 6 ms for T4/T22 and 224 ± 3 ms for T18 (Fig. 3B). This trend was observed for all experimentally fitted peaks, with values ranging from 0.14 – 0.35-fold of the original in vitro value (Table 1).

**Table 1:** Presented here are the globally fitted selT_1_ of the first technical replicate from all three biological replicates, in vitro selT_1_ values and the ratio in vitro / in-cell selT_1_ values, to illustrate the change of selT_1_. Individual fits of each curve are presented in Fig.S2, 3

selT1 [ms]	T8	T9	G20	G6	G11	T4 T22	T18
Global fit technical rep 1	49 ± 13	45 ± 17	101 ± 18		24 ± 25	84 ± 10	
In vitro	331 ± 8	328 ± 6	294 ± 12	286 ± 10	108 ± 2	253 ± 5	224 ± 3
Ratio (in vitro/in-cell)	6.8	7.2	2.9/2.8		4.5	3.1	2.8

### Supernatant preparation can cause artifacts

To control for cell death and sample leakage, it is important to analyse the supernatant surrounding the cells for presence of DNA, which would lead to a non- “in-cell” signal artifact. By comparing two supernatant preparation approaches, we observe that harsher treatment of cells will generate artificial supernatant dsA2 signal. Supernatant experiments were performed on each of the three biological replicates, including a fourth cell preparation utilised for our final SOFAST vs Jump-return comparison. Two approaches to prepare the supernatant NMR sample were explored. The first includes removing the medium and then cell pellet from the Shigemi tube with a Pasteur pipette, and pelleting the cells in an Eppendorf tube, followed by resuspension with a 200 µL pipette and subsequent re-pelleting. The second, and preferred method, instead involves resuspension within the Shigemi by gentle inversion (Fig. 6). Both methods accurately recreate the in-cell sample supernatant by maintaining the total active volume and consisting of the buffer surrounding the cells. In the method where the cell pellet is moved to the Eppendorf tubes and resuspension is achieved by pipetting, larger signals are observed (biological replica 2 and 3 Fig. S1), those prepared according to a more careful protocol with gentle inversion in the Shigemi tube do not (biological replica 1 and the final comparison sample discussed in last Results section).

### Improving 1D-^1^H spectra using measured selective T_1_

**Fig. 7:**
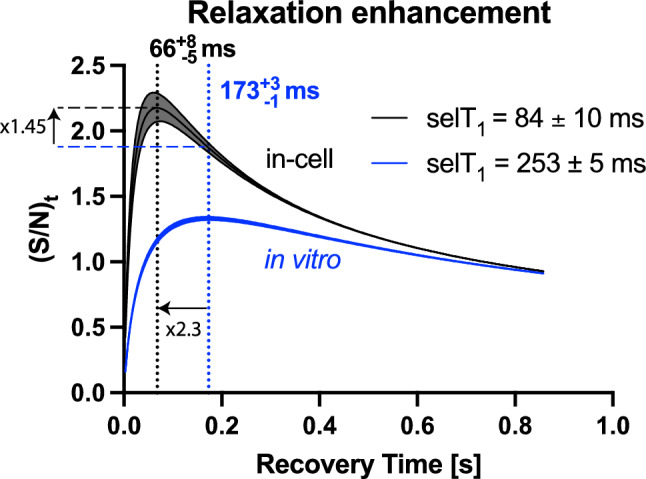
Relaxation enhancement by SOFAST approach. The curve with selT_1_ values indicated, fitted for the slowest recovering peak T4/T22/T18, in-cell (black) and in vitro (blue) using Eq. (1). The optimum interscan delay for the highest sensitivity corresponding to the selT_1_ values are denoted. The standard error is indicated by the shaded region

By using the reduced selT_1_, it was possible to gain signal enhancements for SOFAST-1D experiments, which yielded superior signal-to-noise when compared to delay optimised jump-return spectra. Because the selT_1_ observed is lower than that in vitro*,* this allowed for shortening the recovery time of in-cell SOFAST-1D. Of the many publications concerning the topic of in-cell NMR of nucleic acids, only a few so far use the SOFAST-1D-^1^H approach, including Sakamoto et. al., Broft et. al. and Yamaoki et. al (Yamaoki et al. 2022; Broft et al. 2021a; Sakamoto et al. 2021). Reported 2048 scans require 29 min and 30 s of experimental time (Sakamoto et al. 2021), but acquisition delays, interscan delay, recovery delays and other pulse sequence parameters are not reported for 1D-SOFAST, only for 2D experiments (Broft et al. 2021a). The otherwise used jump-return water suppression scheme for 1D ^1^H experiments requires 14 min 57 s for 1024 scans in our hands, in which the recovery time is set to 1.25*non-selT_1_ (T4/T22 in vitro*)*. As our selT_1_ values above indicate, it is possible to shorten the recovery delay significantly, when using SOFAST experiments in-cell. To estimate the recommended recovery delay for the SOFAST approach (Schanda et al. 2005), one can obtain the interscan delay (D1) recommended for the highest signal intensity by plotting the relaxation enhancement curve (Fig. 7). As a benchmark, the in vitro selT_1_ and its corresponding predicted recovery time maximum was compared with experimentally derived maximum (Fig. S11. The in vitro predicted recovery time gave ${173}_{-1}^{+3}$ ms (upper and lower limits indicated by super- and subscript, respectively) and the experimental value is ${176}_{-13}^{+16}$ ms.

For the slowest recovery time measured, T4/T22/T18, the recommended recovery time for in-cell experiments results in ${66}_{-10}^{+13}$ ms. Figure 7 indicates that the possible signal enhancement is 45% compared to that obtained if the experiment were run with in vitro parameters. However, the recovery time is a combination of the acquisition time, disk write time and recovery delay (D1). A large component of the sequence duration is D1. Default instrument specifications limit the spectrometer D1 to a minimum value of 30 ms, which results in the smallest possible total recovery time of 138 ms in our experimental setup arising from D1 (30 ms) + acquisition time (77 ms) + disk write time (fixed at 30 ms). Through acquiring selT_1_ and without modifying default instrument limitations, we could optimise the relaxation delay to 30 ms which reduced total scan time. The experimental time for our setup was 2 min 37 s for 1024 scans. Using in vitro parameters this time would be 3 min 14 s. This is 1.25 times more scans per unit time with an additional expected sensitivity gain per unit time of 6% (Fig. 7).

For G11, the fastest relaxing in-cell peak, the theoretical enhancement is 82%, but without modifying default instrument limitations would be 19%, if comparing in vitro experiments chosen for maximal G11 sensitivity. If the goal is to maximise signal-to-noise for all visible peaks, the possible enhancement would be 24% if the parameters are compared to those chosen as optimal for all iminos in-cell and in vitro, which would be that of the slowest recovering peak T4/T22/T18.

These are considerations for the fast-pulsing regime, where magnetisation does not fully recover to equilibrium before the next scan begins. This means that the experiments are semi-quantitative. In order to perform quantitative experiments, full magnetisation recovery is required, typically a value of 5 × T_1_ is used. Here, SelT_1_ optimised experiments enable a more significant decrease in scan time. For T4/T22/T18 the benefit in the quantitative regime is substantial, with 22 min 27 s for the unoptimised experiment and 7 min 5 s for the optimised experiment. This corresponds to 3 times more scans per unit time with an expected sensitivity gain of 70%.

### In-cell dsA2 recovery time optimised ^1^H-Jump-Return versus ^1^H-imino-SOFAST

**Fig. 8:**
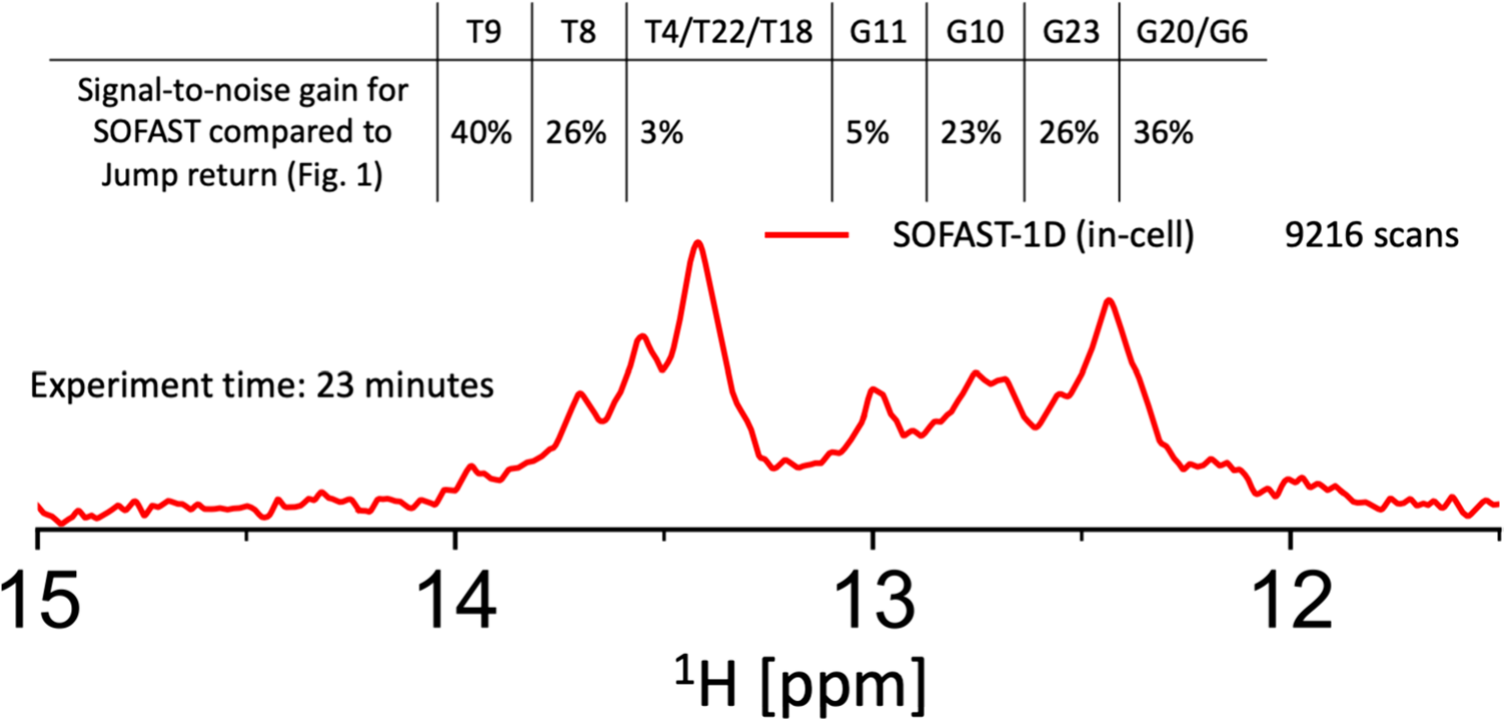
A quantitative comparison of the SOFAST pulse program (red spectrum) with the Jump-Return echo sequence seen in Fig. 1 (orange spectrum) on the same sample. These ^1^H spectra of dsA2 in HeLa cells were recorded in 23 min 39 s (9216 scans) and 22 min 29 s (1024 scans), respectively

As a final proof-of-concept the recovery-optimised SOFAST was compared to a recovery-optimised jump-return, which was measured based on in vitro parameters (Fig. 1, Fig. 7). An improvement in acquisition speed and signal-to-noise is observed when the measured selT_1_ in-cell is used to optimize the SOFAST experiment, in comparison with the jump-return spectra, where parameters were optimised in vitro. The two experiments were performed on the same sample, where first the in vitro optimized jump-return, and then the in-cell optimized SOFAST were performed both with the same total experimental time Fig. 8. The frequently used jump-return water suppression scheme for 1D ^1^H experiments requires 23 min for only 1024 scans in our hands, in which the recovery time is set to 1.3 s, which is 1.3–6 times the non-selective T_1_ in vitro. The optimal recovery time for 90 degree fast-pulsing regime is 1.25*T_1_. In this case the recovery time is 1.3*nonselT_1_ of T9, but 1.9 times for T4/T22, and 6-times for the fastest peak G11. The in-cell delay-optimised SOFAST experiments allowed us to record 9216 scans within the same time, resulting in a 40% higher signal-to-noise (8.14 to 11.4 S/N) for the second fastest relaxing signal (T9), and 4% for the slowest (T4/T22/T18) (Figs. 1C).

## Discussion

### Selective longitudinal relaxation time and sensitivity gain

Speeding up measurement time and increasing signal-to-noise are crucial for in-cell NMR, especially of less stable nucleic acids. However, they rarely work in tandem. The SOFAST experiment offers both, and by further optimising the recovery delay the maximum possible sensitivity for a sample can be achieved (Schanda et al. 2005). To optimise the recovery delay, the in-cell and in vitro selT_1_ were determined by measuring a series of selective inversion recovery experiments. It was observed that the selT_1_ was 2.8–7.2 (for the different imino base-pairs) times higher in vitro than in-cell. This reduction in selT_1_ may be attributed to several factors including reduced molecular tumbling, which has been observed for proteins in cell (Luchinat and Banci 2017; Barbieri et al. 2015). The selective T_1_ is inversely correlated to tumbling for biomacromolecules (Schanda et al. 2005; Tinoco and Figueroa-Villar 1999). Increased base-pair opening kinetics can also contribute to the decreased selT_1_, as k_ex_ was shown to increase ~ tenfold for telomeric DNA in cell (Yamaoki et al. 2022). Free paramagnetic species may also reduce selT_1_ (Wan et al. 2023; Marasini et al. 2021; Bernsen et al. 2010). This reduction of selT_1_ in the cell allowed for optimised parameters to be used for the in-cell NMR experiment, which allowed for faster acquisition than in vitro. This resulted in a sensitivity gain of individual imino proton signals ranging from 2–40% for the in-cell SOFAST experiment in comparison to the jump-return experiment. Two-dimensional experiments are rarely applied in nucleic acid in-cell NMR of human cells, due to long experimental time (Hänsel et al. 2009; Broft et al. 2021a), however, implementing the SOFAST approach with optimised selT_1_ and the following shortened recovery delay could facilitate the use of more 2D experiments in the field. Furthermore, this approach is also of advantage for time-resolved kinetic NMR studies and allows for a higher time resolution on top of better S/N, as well as for any studies in which the fragility of the cells is increased (Luchinat et al. 2020).

Alternative routes exist to determine the optimal recovery time for the SOFAST experiment, including determining it by running SOFAST spectra with varying recovery times (Fig. S11), and this approach can be used to determine selT_1_ by fitting the curve to Eq. (1). However, this method is, for example, not suitable to determine short selT_1_ values, where the total recovery time is shorter than that permitted by the instrument. The experimentally determined selT_1_ has several other possible applications. It can be applied for the determination of water exchange to study base pair openings (Yamaoki et al. 2022) as well as alongside non-selective T_1_ to estimate tumbling times (Tinoco and Figueroa-Villar 1999).

### Supernatant control

Several experimental controls are important during an in-cell NMR experiment, including supernatant measurement, subcellular localisation, cellular viability, and cellular quantification. The experimental procedures of transfection and the following compaction into the small space of the NMR tube are known to cause leakage of DNA/RNA and proteins from the cells (Yamaoki et al. 2018; Mu et al. 2017), as well as the supernatant procedure itself, as we show here (Mu et al. 2017). We assume that diffusion of metabolites or DNA throughout the NMR tube is not instantaneous and complete, resulting in concentration gradient over the length of the NMR sample tube. Therefore, the extraction and analyses of a control sample representing the supernatant has to be carefully designed to reflect the exchange of cells with the medium around the cell pellet (Barbieri et al. 2016, 2015). By comparing supernatant procedures, we suggest an optimised protocol for the control of the supernatant. Accordingly, our protocol promotes a gentle resuspension and pelleting of cells within the Shigemi tube to acquire the supernatant control. As we, like others, observed that coarse treatment, such as twofold washing or shearing by pipetting, leads to increased cell death and leakage (Fig. S1) (Mu et al. 2017).

### Quantification of cellular nucleic acid

The NMR signals are proportional to the concentration of nucleic acid present in the sample, and we therefore optimised the protocol to accurately determine the concentration of DNA transfected into the cell (Sakamoto et al. 2021). This is important to be able to draw conclusions of biological effects correlating to the signal. First, two wash-volumes directly after transfection were used, as with a singular wash we have estimated that we can expect 2.8 µM extracellular DNA in our in-cell NMR sample, due to the volume of medium retained in a cellular pellet. To estimate the concentration of introduced DNA in the cell, we used a simple but robust extraction of the transfected DNA spiked with 2.5% fluorophore-labelled DNA. This can be compared to the measured signal to estimate the visible liquid-state NMR fraction. Currently used fluorescent labels for control experiments have drawbacks including environment specific intensity, structural and / or trafficking alterations. Therefore one could use Northern hybridisation instead for gel quantification, or explore FISH experiments (Streit et al. 2009) to avoid these labels entirely (Viles et al. 2001).

Our data also indicates that a quantitative evaluation of the signal localisation is necessary. The concentration of dsA2 is visibly higher in the nucleus, in line with previous research (Yamaoki et al. 2018, 2022; Dzatko et al. 2018; Krafčík et al. 2021; Sakamoto et al. 2021), however, only upon considerations of nuclear vs cytoplasmic volumes, it is clear that at a minimum almost half of the species present inside the cell are in the cytoplasm (70 ± 29%) (Fig. S2).

### Biological replicates and reproducible data processing

Depending on the biological research question, it is important to consider the different cell states present in different NMR samples. This highlights the necessity of biological replicates when attempting to quantify cellular parameters. Several biological replicates are standard in the field of biology, but not yet in structural biology or structural NMR (Müntener et al. 2016; Miccheli et al. 2006; Bines et al. 1985). Here we employ biological replicates as well as multiple data-processing procedures to extract the intensity of the peaks, which, given the low S/N of in-cell NMR experiments, is a needed internal control for interpretability of the data.

## Summary

Through modifications both to the acquisition and analysis of existing control experiments, alongside employing biological replicates, we are able to determine the lower selT_1_ value inside the cell. Here, we applied the measured selT_1_ to optimise the parameters, namely the recovery delay, of 1D-SOFAST experiment and obtain maximum sensitivity higher than that of the jump-return experiment, and according to in vitro benchmarks, also higher than that of the unoptimized SOFAST. This methodology can be applied to any in-cell nucleic acid or protein system and should facilitate more complex in-cell NMR experiments, including those to elucidate e.g. base pair opening rates, estimate tumbling times or reveal physico-chemical effects of the cell on nucleic acids.

## Supplementary Information

Below is the link to the electronic supplementary material.

Supplementary file1 (TIF 55258 KB)

Supplementary file2 (DOCX 27657 KB)

Supplementary file3 (XLSX 20 KB)

## Data Availability

The authors confirm that the data supporting the findings of this study are available within the article [and/or] its supplementary materials. Scripts and raw NMR data are available at github.com/petzoldlab/T1-in-cell.
