# Supplementary figures and images for: Optimising in-cell NMR acquisition for nucleic acids
Source: J Biomol NMR. 2024 Aug 20;78(4):249–64. doi: 10.1007/s10858-024-00448-5 (PMC11614993; doi:10.1007/s10858-024-00448-5)

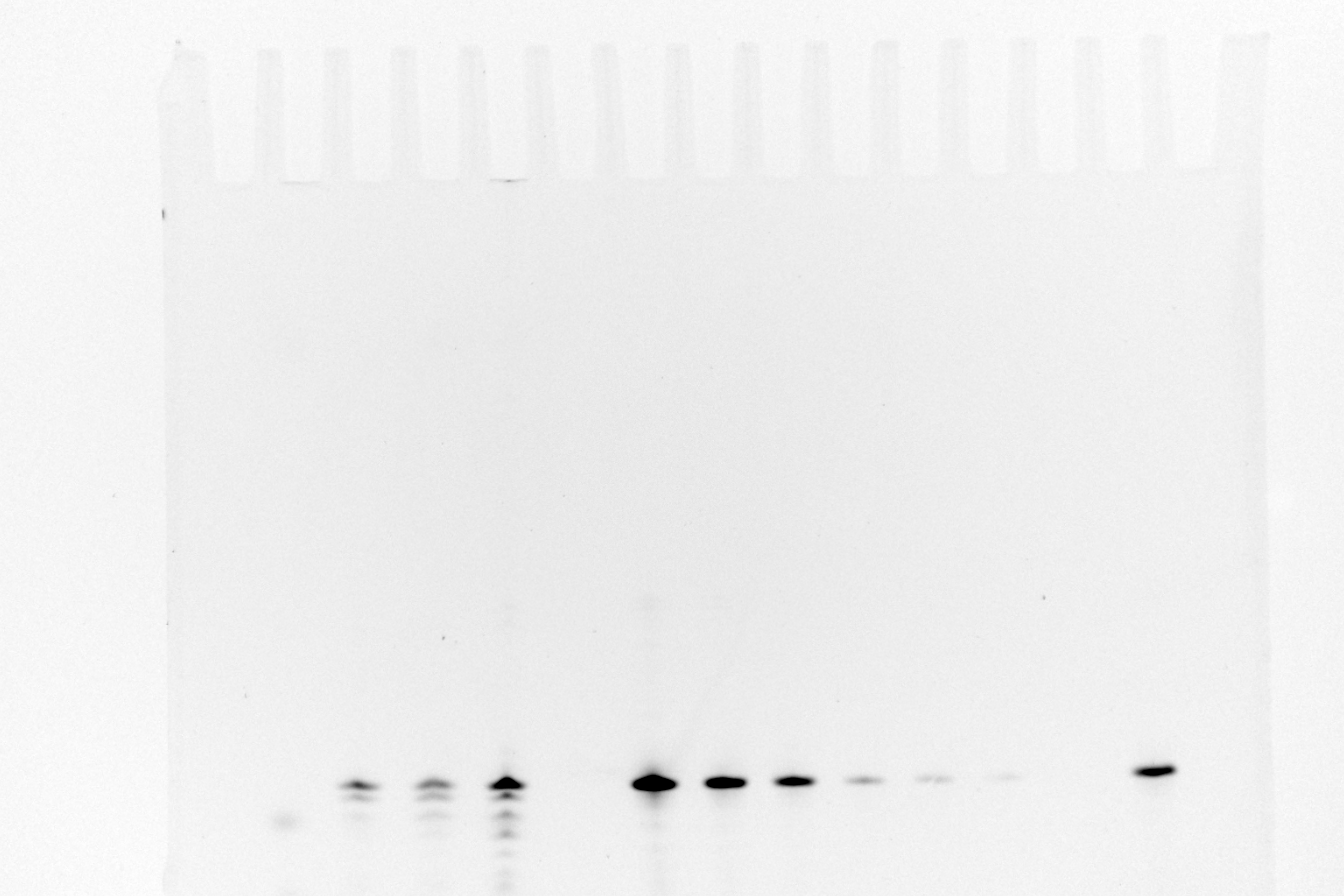

Supplement: Supplementary file 1 — Supplementary file1 (TIF 55258 KB) [file 10858_2024_448_MOESM1_ESM.tif]
