# Supplementary material for: Optimising in-cell NMR acquisition for nucleic acids
Source: J Biomol NMR. 2024 Aug 20;78(4):249–64. doi: 10.1007/s10858-024-00448-5 (PMC11614993; doi:10.1007/s10858-024-00448-5)
Supplement: Supplementary file 2 — Supplementary file2 (DOCX 27657 KB) [file 10858_2024_448_MOESM2_ESM.docx]

Supplementary Information

**Optimising In-cell NMR Acquisition for Nucleic Acids**

Henry T. P. Annecke^1,2^ & Reiner Eidelpes^1^, Hannes Feyrer^1^, Julian Ilgen^1^, Cenk Onur Gurdap^3,4^, Rubin Dasgupta^2^, Katja Petzold^1,2,4,5,*^

^1^Department of Medical Biochemistry and Biophysics, Karolinska Institutet, Solnavägen 1, 171 65 Stockholm, Sweden

^2^Department of Medical Biochemistry and Microbiology, Biomedical Center, Uppsala University, Husargatan 3, 752 37 Uppsala

^3^Department of Women’s and Children’s Health, Karolinska Institutet, 171 65 Solna, Sweden

^4^Science for Life Laboratory, 171 65 Solna, Sweden

^5^Center of Excellence for the Chemical Mechanisms of Life, Uppsala University, 752 37 Uppsala, Sweden

*Corresponding author: Katja Petzold (katja.petzold@imbim.uu.se)

Supplementary Table 1: **Viability as determined by trypan blue staining and flow cytometr**y. n=3 (± 1 standard deviation.) unless indicated with * for which n =1

|  | Viability (Electroporation) | | | Transfected and Viable |
| --- | --- | --- | --- | --- |
|  | Before | After | 4 hours after |  |
| Flow cytometry | 97 ± 4 % | 89 ± 9 % |  | 84 ± 11 % |
| Trypan Blue | 82 ± 4 % | 77 ± 6 % | 69 % * |  |


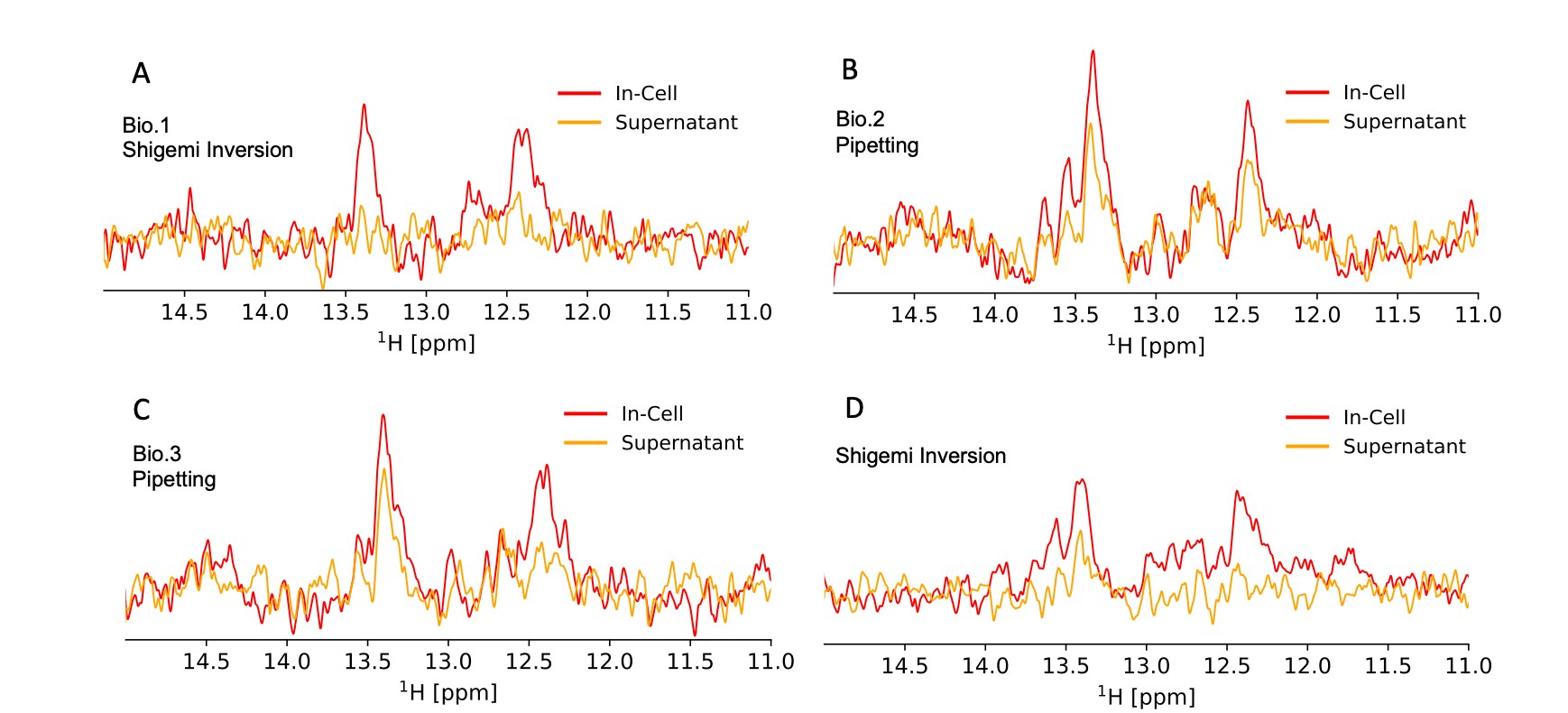


Supplementary Figure 1: **Initial sample control using 1D-SOFAST 1024 scans before inversion recovery experiments alongside supernatant controls following first technical replicate.** Biological replicate 1, B: Biological replicate 2, C: Biological replicate 3, D: final HeLa sample used in SOFAST vs jump-return experiment.


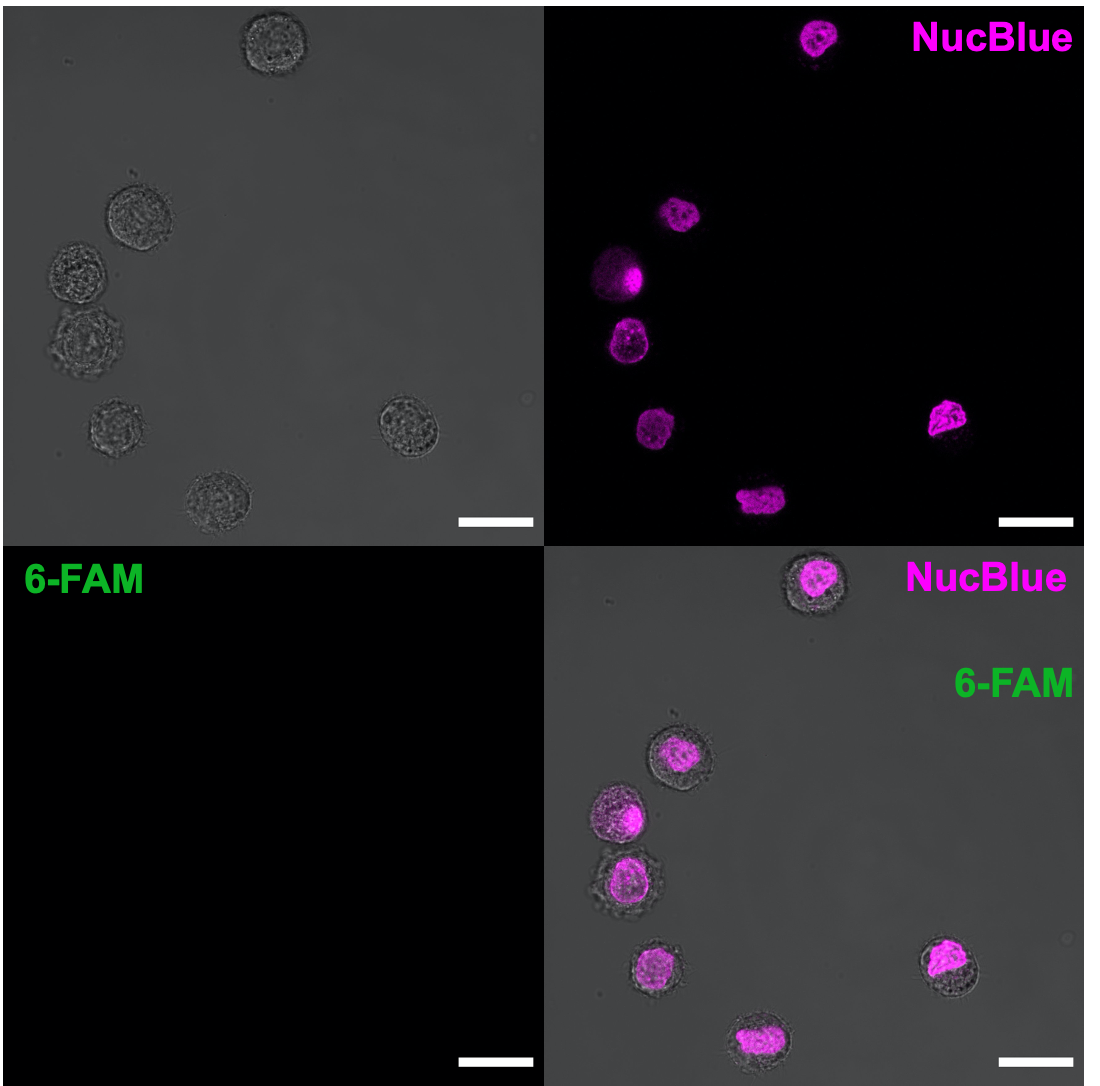

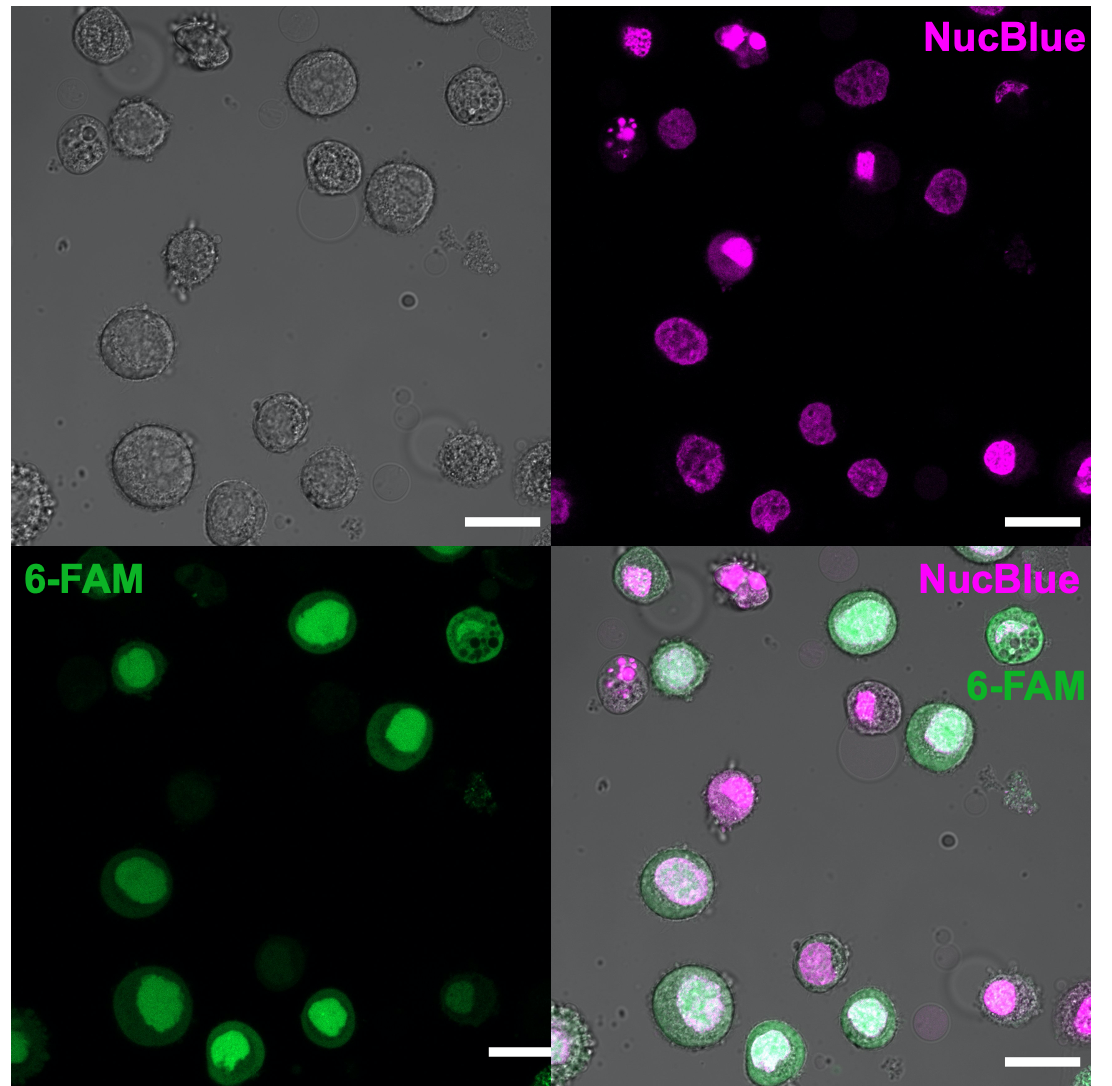

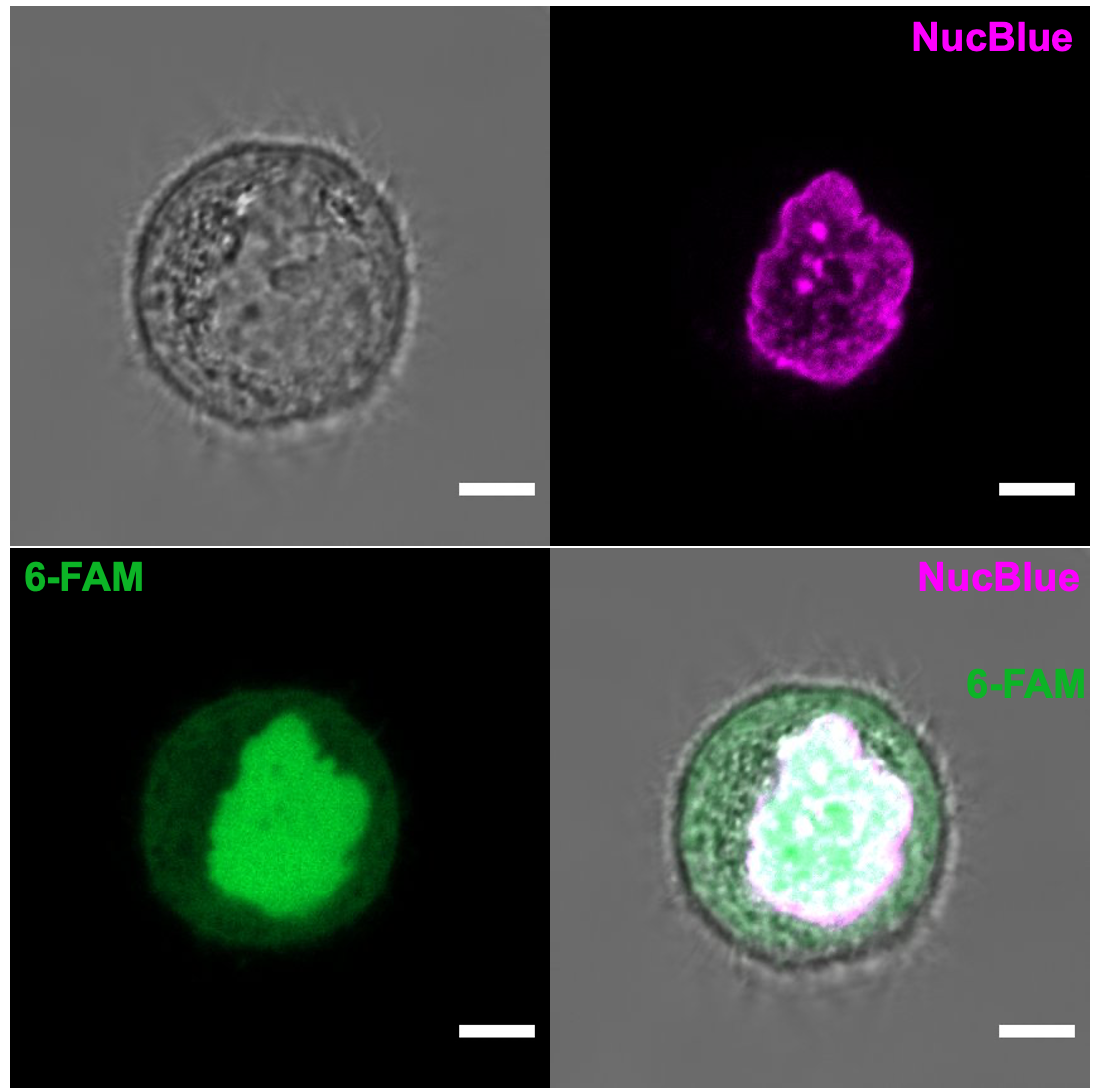


C

B

A

Supplementary Figure 2: **Confocal microscopy images of in-cell sample preparation**. Showing: top left brightfield, top right NucBlue nuclei stain (violet), bottom left 6-FAM dsA2 (green), bottom right overlay of all three with A) unelectroporated control B) electroporated cells C) close up of electroporated HeLa. Scale bar represents 20 µm in first two images, and 5 µm in final image.

Supplementary Figure 3: **Standard curve of the analyzed gel presented in Figure 2.** Left: Indicated in crosses are the biological replicates. Right: Data including 0.5 pmol data point, which displays non-linear behaviour. The most plausible explanation is that the 0.5 pmol data point saturates the detector and hence is outside the dynamic range leading to a lower estimation in concentration. The cell samples lie within the linear range and so a linear fit is used.


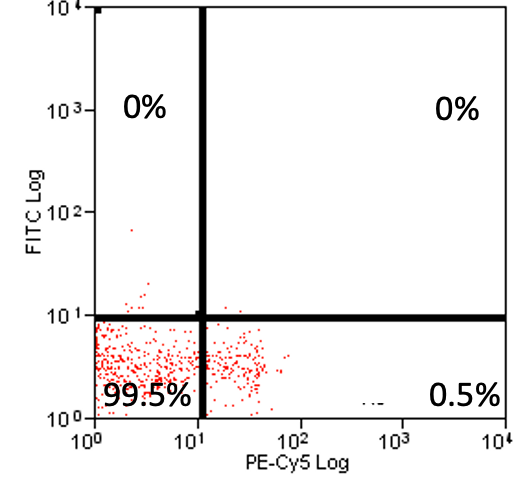

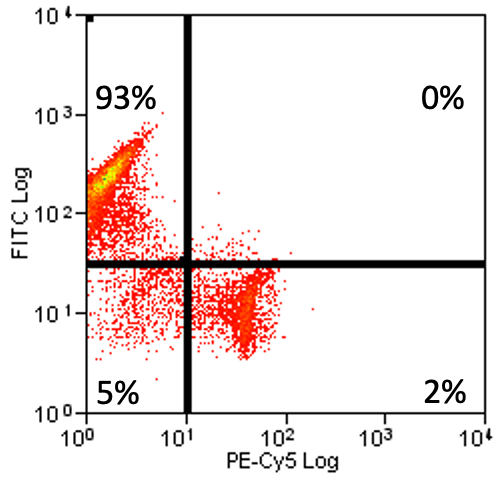


Supplementary Figure 4: **Example flow cytometry results from biological replicate 1 cells stained with 7AAD to assess viability and presence of dsDNA target before and after electroporation**[1]**.** Images are split into quadrants, with top-left indicating samples viable and transfected, top right non-viable and transfected, bottom left viable and untransfected, bottom right non-viable and untransfected.


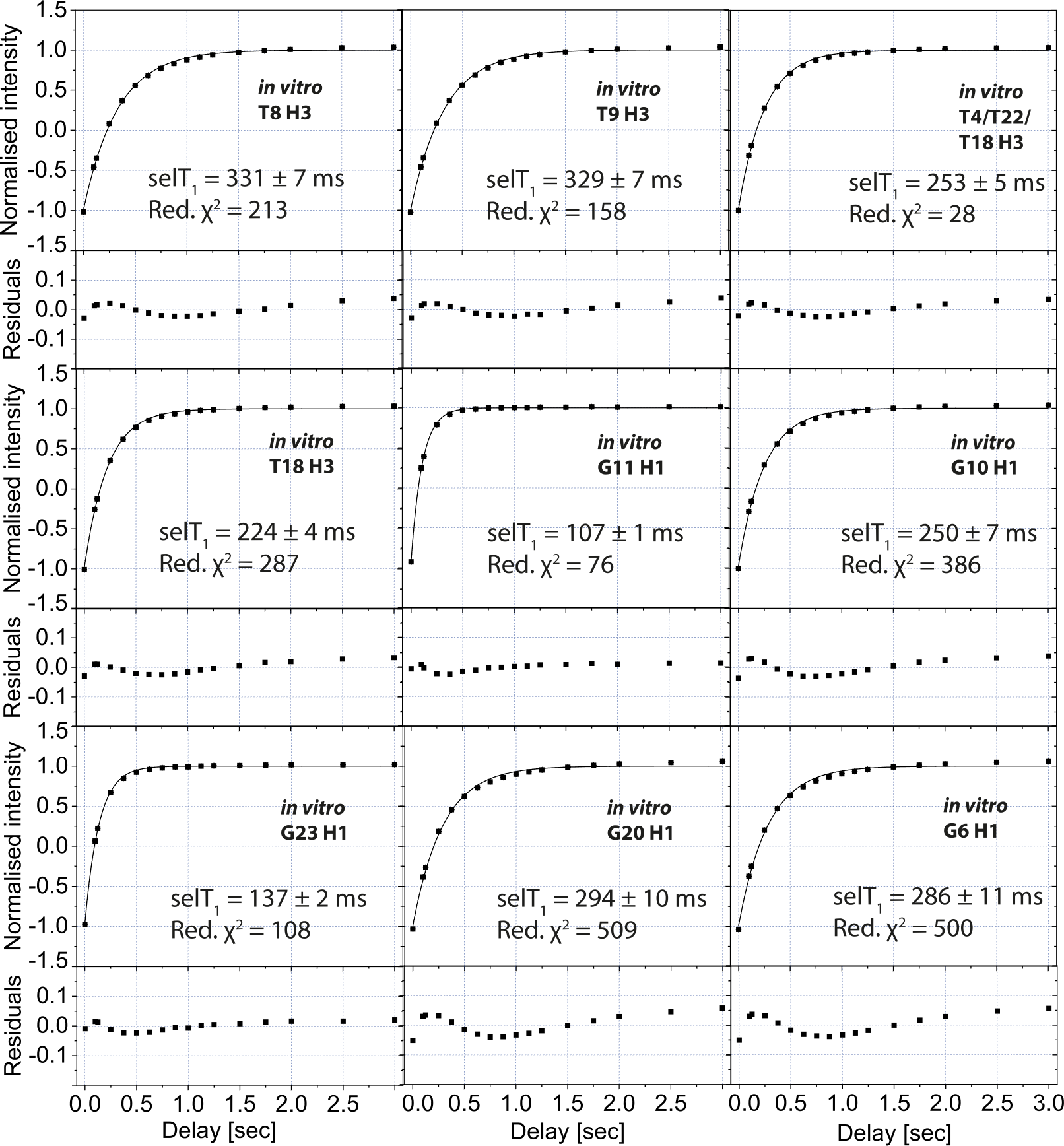


Supplementary Figure 5: **Individual fits for in vitro replica with equation I = A*exp(-t/T1) + D for data analysed with line broadening.**


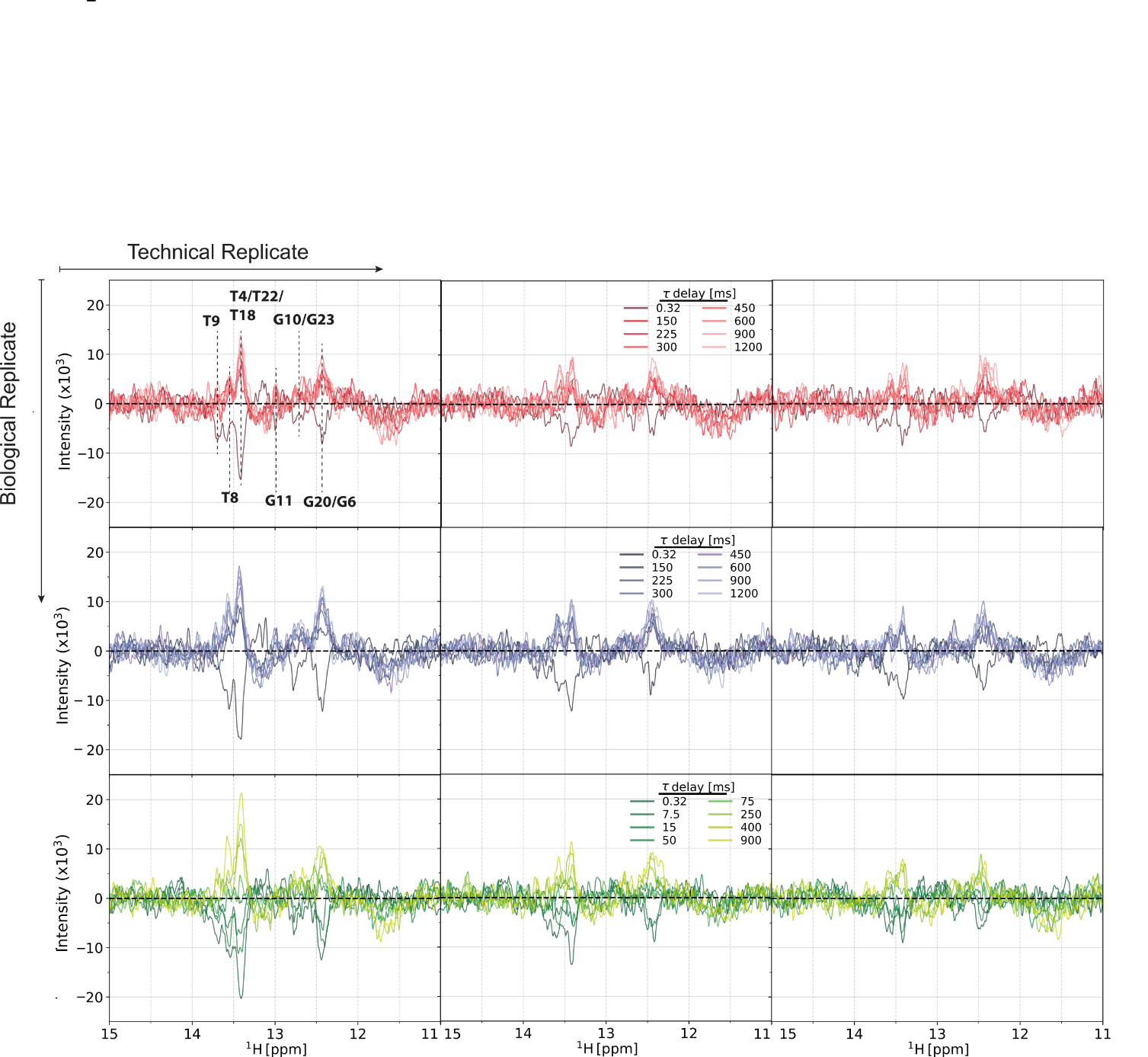


Supplementary Figure 6: Overview of all replica measured processed with 15 Hz line broadening. Top row represents the first biological replicate, top left the first technical replicate, top middle the second technical, top right the third technical. Middle row is second biological replicate, and final row is third biological replicate.

Supplementary Table 2: Fitted selT_1_ values for fitable individual in-cell. Dashes indicate fits that did not converge or failed the ANOVA test for significance (SI Figure 8).

| **selT_1_ [ms]** |  | **T8** | **T9** | **G20 G6** | **G11** | **T4 T22** |
| --- | --- | --- | --- | --- | --- | --- |
| Biological replicate 1 | Technical rep. 1 | - | - | 147 ± 27 | 41 ± 75 | 117 ± 17 |
|  | Technical rep. 2 | - | - | 107 ± 47 | - | 159 ± 44 |
|  | Technical rep. 3 | - | - | 250 ± 73 | - | 178 ± 26 |
| Biological replicate 2 | Technical rep. 1 | 165 ± 30 | 262 ± 138 | 142 ± 9 | - | 111 ± 17 |
|  | Technical rep. 2 | 65 ± 28 | - | 132 ± 36 | - | 158 ± 35 |
|  | Technical rep. 3 | - | - | 137 ± 40 | - | 150 ± 60 |
| Biological replicate 3 | Technical rep. 1 | 44 ± 10 | 46 ± 12 | 45 ± 49 | 6 ± 4 | 70 ± 11 |
|  | Technical rep. 2 | 32 ± 5 | - | 51 ± 22 | - | 47 ± 11 |
|  | Technical rep. 3 | 47 ± 12 | - | 103 ± 19 | - | 80 ± 11 |


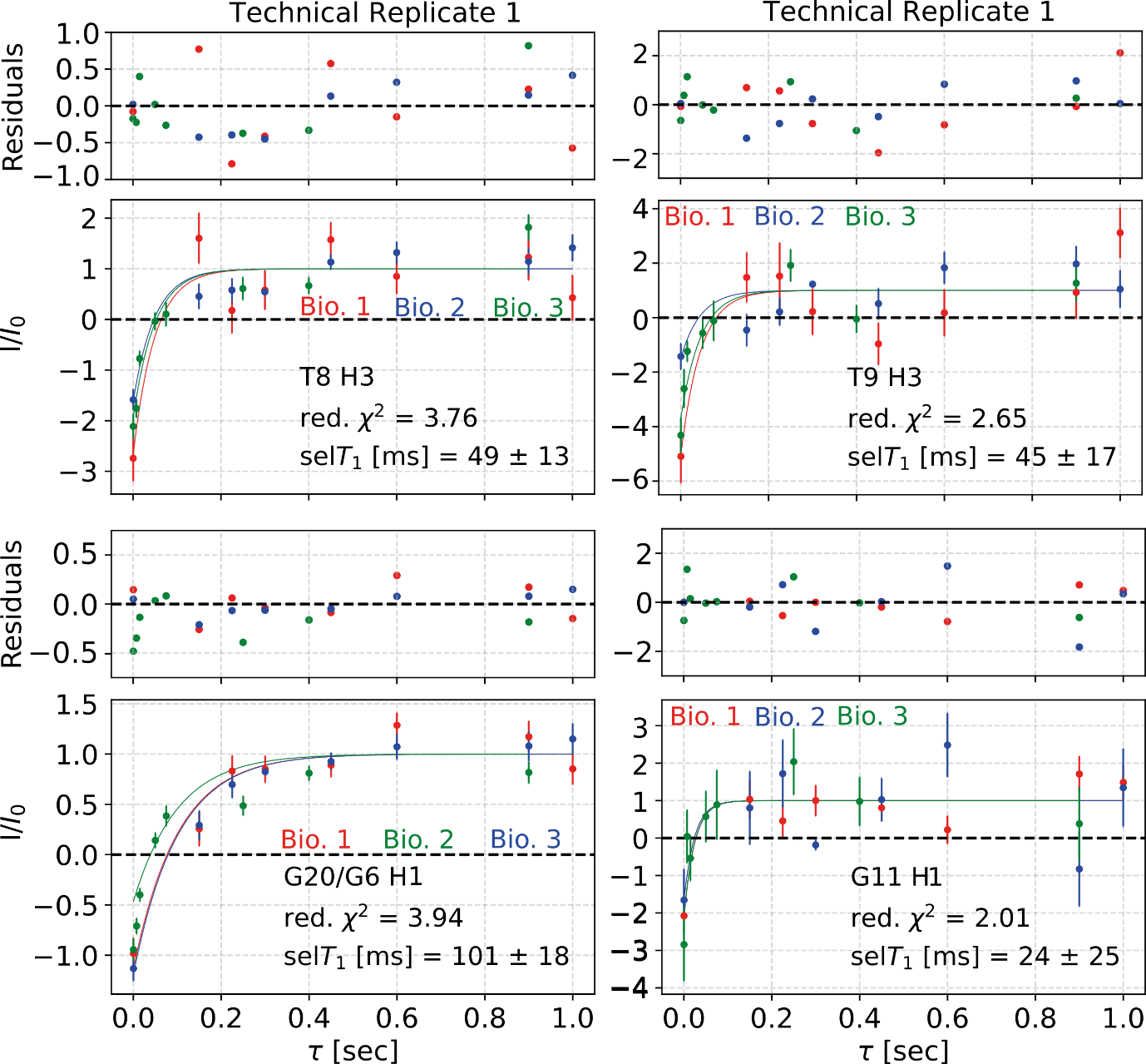


Supplementary Figure 7: **Global fits with shared parameter selT_1_ for in-cell selective inversion recovery remaining imino peaks of the first technical replica between all three biological replica**. Fitted to equation I/I0 = Aexp(-t/selT1) + D. Intensities were normalised to signal intensity at equilibrium.


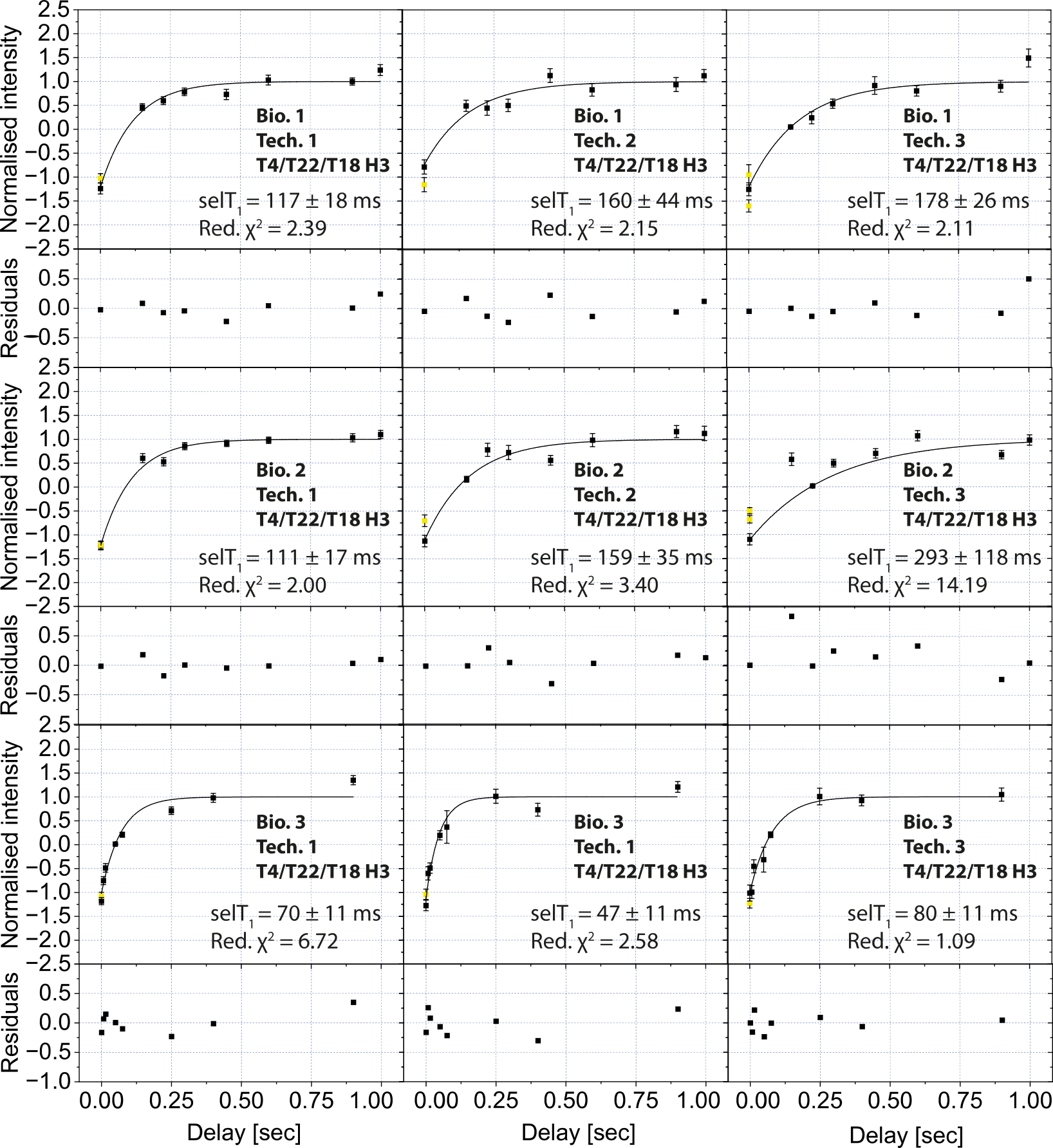

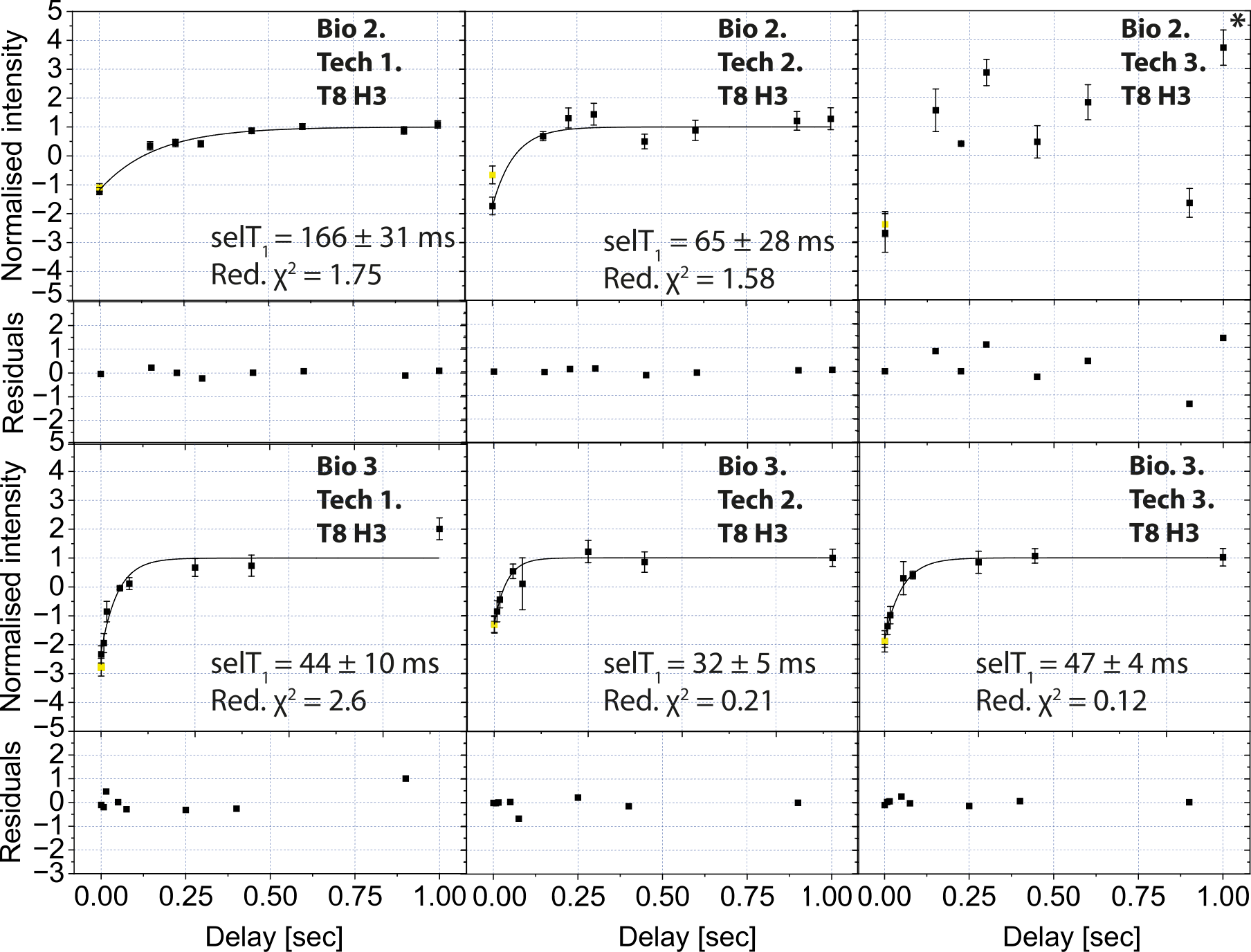

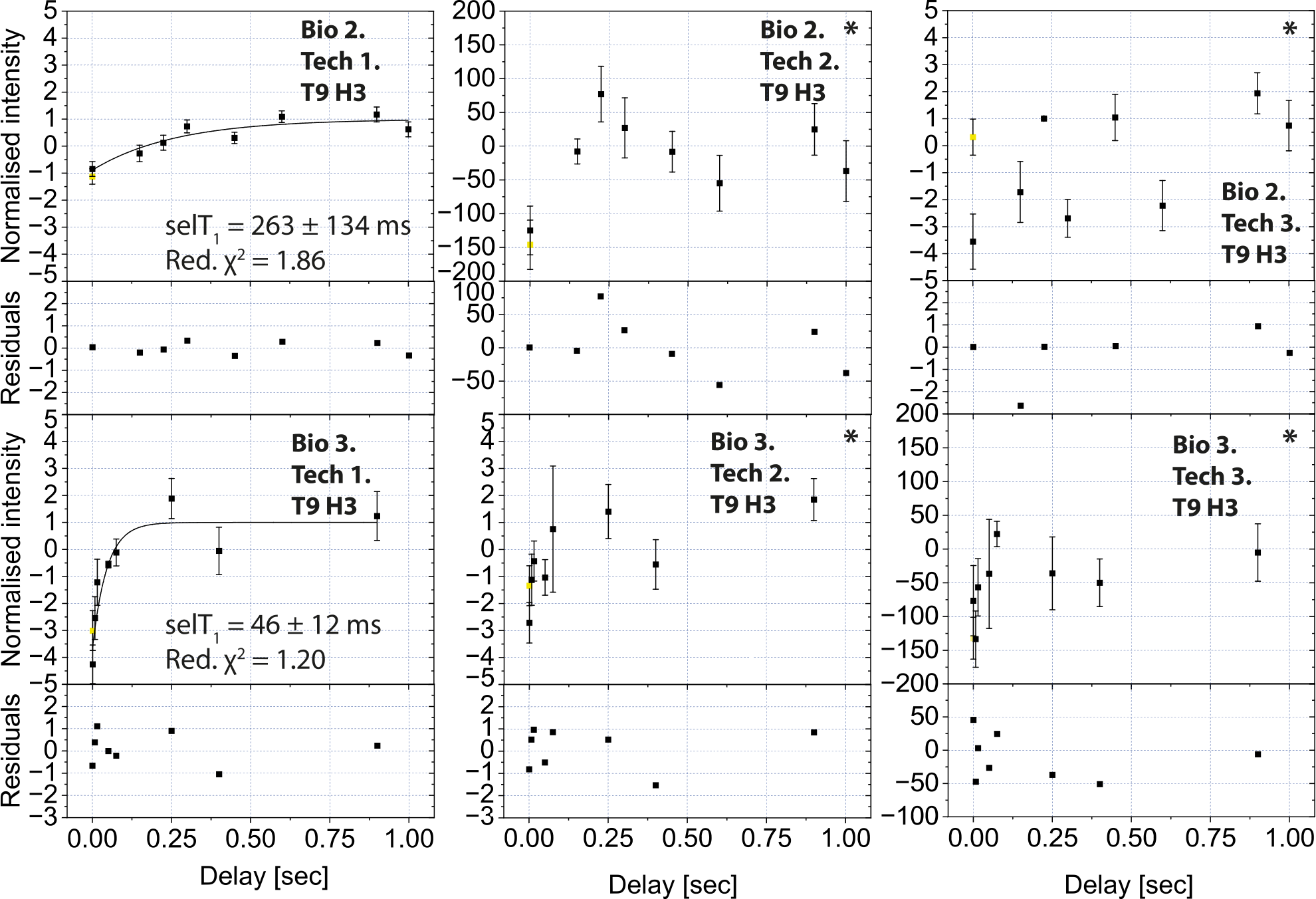

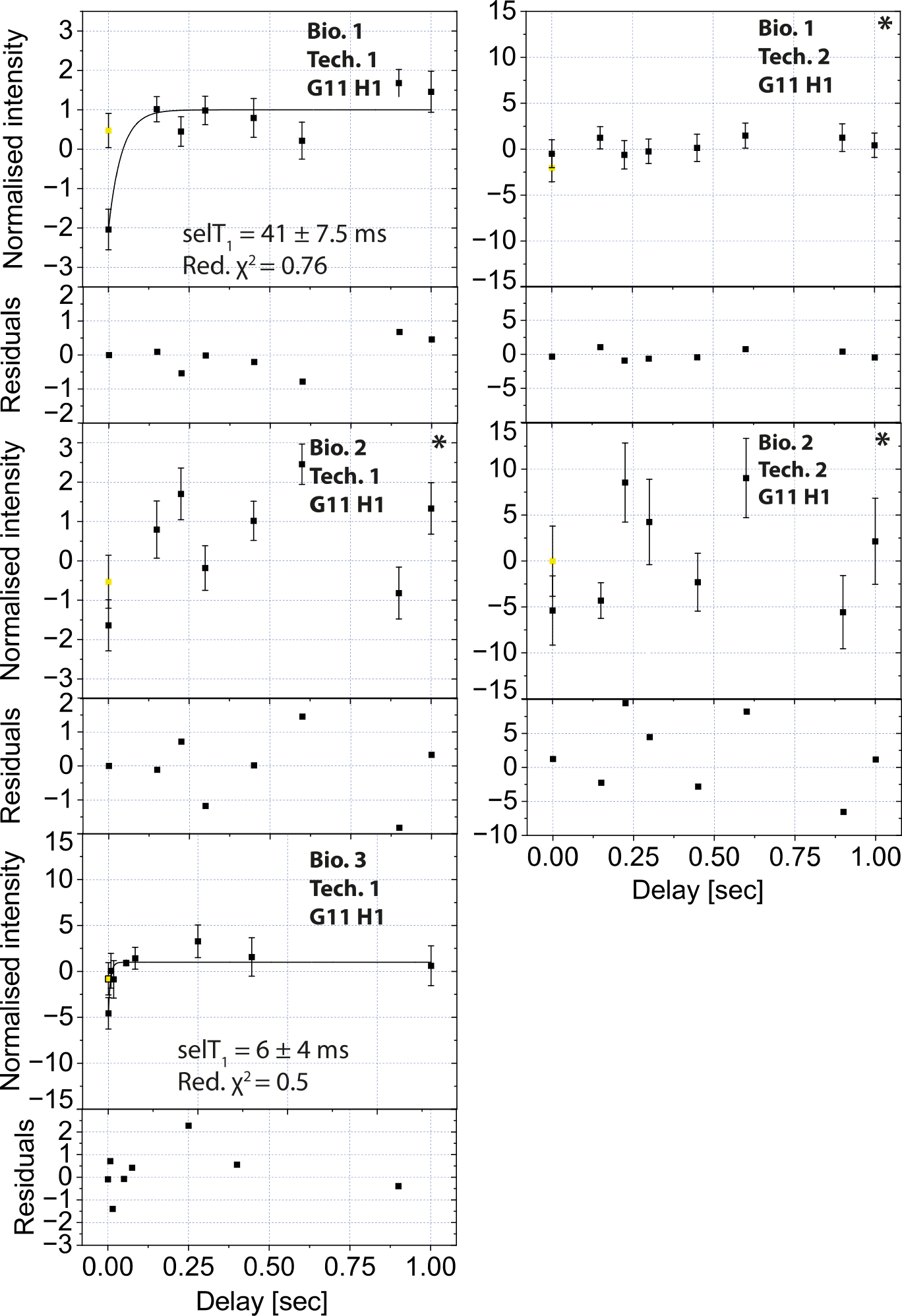

Supplementary Figure 8: **Individual fits for in-cell replica with equation I = A*exp(-t/T1) + D for data analysed with line broadening.** Points in yellow represent the repeated 0.32 ms delay point used to estimate sample degradation over the experimental time span. These are excluded from fitting. Data with * indicated fail ANOVA test for significance. Fits with no convergence are not shown.

*
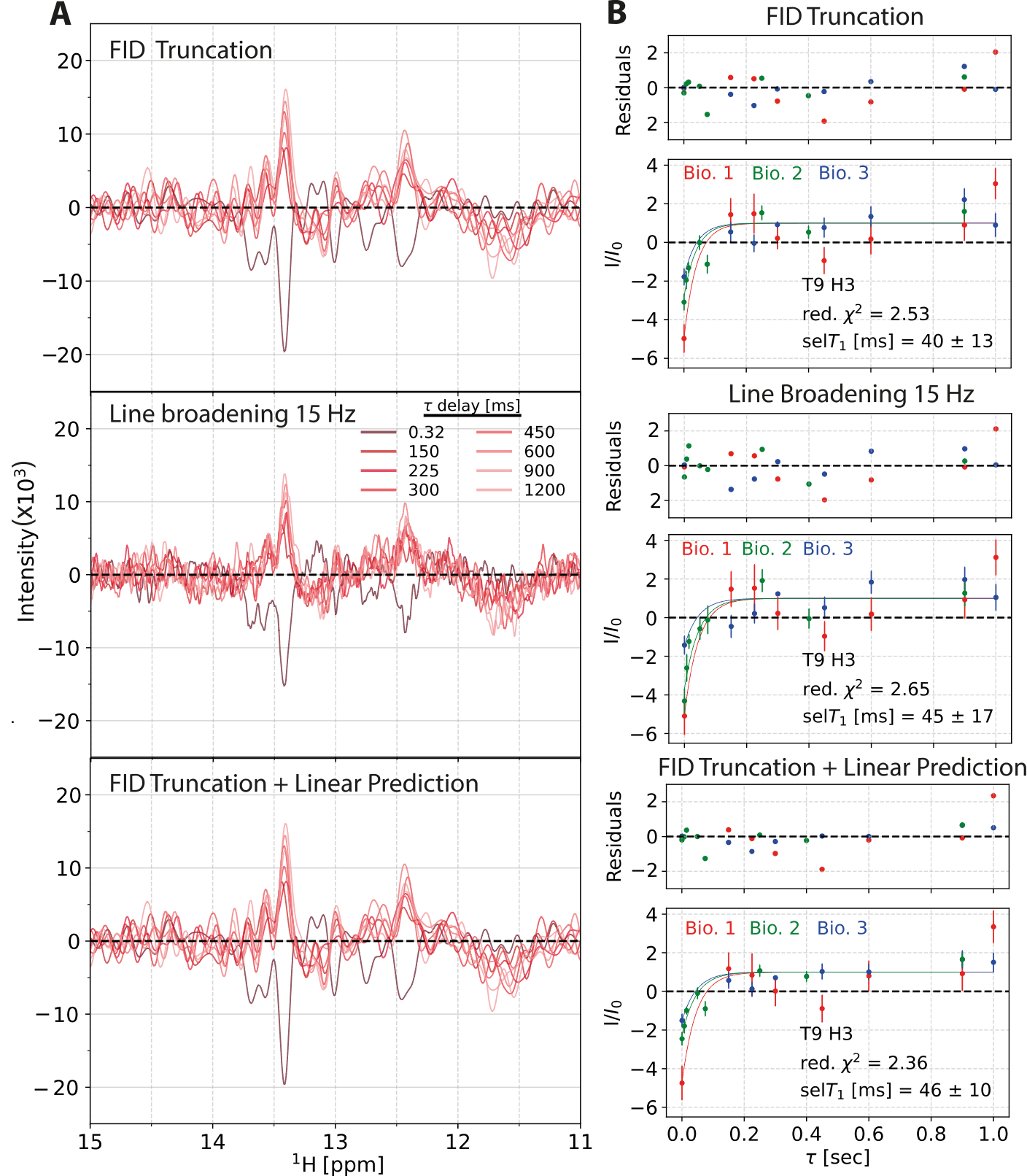
*

Supplementary Figure 9: **Global fits sharing the parameter selT_1_ of T9 using each processing method.** A: Sample spectra from in-cell biological replicate 1. Top – FID Truncation, middle - with line broadening, bottom – FID Truncation with linear prediction. B: Respective global fits from all biological replicates and the effect on the fitted parameters.

*
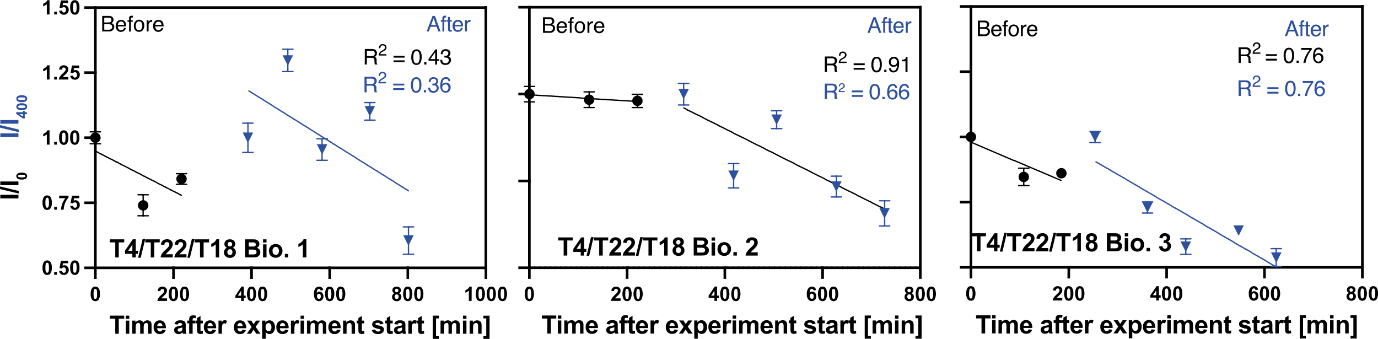
*

Supplementary Figure 10: **Sample degradation due to DNA degradation and cell death.** Each point represents a repeated 0.32 ms experiment. Linear fits for the correction factor for biological replicate 1, 2, and 3. Normalisation is repeated following supernatant treatment, represented as Before (black), and After (blue). Error bars are within the data points for Bio. 3. Fit values in Table S2.

Supplementary Table 2**: Signal deterioration fits from linear regression of repeated inversion experiments using the same inversion recovery delay time (0.32 ms) (Figure SI 3**). First row represents percentage decay per hour for first technical replicate (-4.6 ± 5.6 %/hr for Bio. Rep. 1), second row represents signal decay after supernatant preparation and repelleted sample (-5.4 ± 4.3 %/hr for Bio. Rep. 1)

| Experiment | Signal Decay |
| --- | --- |
| Bio. Rep. 1 | - 4.6 ± 5.6 %/h  - 5.4 ± 4.3 %/h |
| Bio. Rep. 2 | - 1.2 ± 0.3 %/h  - 8.5 ± 2.1 %/h |
| Bio. Rep. 3 | - 4.7 ± 2.6 %/h  - 6.5 ± 2.1 %/h |

Supplementary Figure 11: **Experimental results for optimal recovery time of in vitro 1D-SOFAST**. 1D-SOFAST were collected with varying D1 delays. Shaded lines represent 95% confidence interval. Vertical line indicates maximum point from fit.

References

[1] I. Schmid, W.J. Krall, C.H. Uittenbogaart, J. Braun, J. V. Giorgi, Dead cell discrimination with 7‐amino‐actinomcin D in combination with dual color immunofluorescence in single laser flow cytometry, Cytometry 13 (1992). https://doi.org/10.1002/cyto.990130216.
